# A Double-Blind Randomized Phase I Clinical Trial Targeting ALVAC-HIV Vaccine to Human Dendritic Cells

**DOI:** 10.1371/journal.pone.0024254

**Published:** 2011-09-16

**Authors:** Michael A. Eller, Bonnie M. Slike, Josephine H. Cox, Emil Lesho, Zhining Wang, Jeffrey R. Currier, Janice M. Darden, Victoria R. Polonis, Maryanne T. Vahey, Sheila Peel, Merlin L. Robb, Nelson L. Michael, Mary A. Marovich

**Affiliations:** 1 U.S. Military HIV Research Program (MHRP), Rockville, Maryland, United States of America; 2 International AIDS Vaccine Initiative (IAVI), New York, New York, United States of America; 3 Walter Reed Army Institute of Research, Silver Spring, Maryland, United States of America; University of Toronto, Canada

## Abstract

**Background:**

We conducted a novel pilot study comparing different delivery routes of ALVAC-HIV (vCP205), a canarypox vaccine containing HIV gene inserts: *env*, *gag* and *pol*. We explored the concept that direct *ex vivo* targeting of human dendritic cells (DC) would enhance the immune response compared to either conventional intramuscular or intradermal injections of the vaccine alone.

**Methodology/Principal Findings:**

Healthy HIV-1 uninfected volunteers were administered ALVAC-HIV or placebo by intramuscular injection (IM), intradermal injection (ID) or subcutaneous injection (SQ) of autologous *ex vivo* transfected DC at months 0, 1, 3 and 6. All vaccine delivery routes were well tolerated. Binding antibodies were observed to both the ALVAC vector and HIV-1 gp160 proteins. Modest cellular responses were observed in 2/7 individuals in the DC arm and 1/8 in the IM arm as determined by IFN-γ ELISPOT. Proliferative responses were most frequent in the DC arm where 4/7 individuals had measurable responses to multiple HIV-1 antigens. Loading DC after maturation resulted in lower gene expression, but overall better responses to both HIV-1 and control antigens, and were associated with better IL-2, TNF-α and IFN-γ production.

**Conclusions/Significance:**

ALVAC-HIV delivered IM, ID or SQ with autologous *ex vivo* transfected DC proved to be safe. The DC arm was most immunogenic. Proliferative immune responses were readily detected with only modest cytotoxic CD8 T cell responses. Loading mature DC with the live viral vaccine induced stronger immune responses than loading immature DC, despite increased transgene expression with the latter approach. Volunteers who received the autologous vaccine loaded mature DC developed a broader and durable immune response compared to those vaccinated by conventional routes.

**Trial Registration:**

ClinicalTrials.gov NCT00013572

## Introduction

The HIV pandemic remains largely unchecked with massive morbidity, mortality and social instability consequences. Current estimates indicate about 60 million people have been infected worldwide, with Africa incurring the greatest number (70%) of global infections [1]. It is increasingly apparent that controlling this disease will require unprecedented measures from the scientific field, especially with regards to the development of an efficacious vaccine [2]. To date, nearly 200 clinical trials have been conducted with potential vaccine candidates to prevent or treat HIV infection; one-quarter of these trials explored the use of pox-based viral vectors [3]. ALVAC, a host range restricted canarypox virus, is replication incompetent in humans and considered safe. Recently, ALVAC has shown modest efficacy in preventing HIV acquisition in the ALVAC-HIV/AIDSVAX B/E Phase III trial conducted in Thailand (RV144) [4]. Despite the encouraging outcome, immune responses with ALVAC need further understanding and optimization.

A relatively novel vaccine strategy incorporates reinfusion of autologous vaccine loaded dendritic cells (DC). Since their discovery in 1973, a growing body of literature identifies the central role of DC in antigen processing, presentation and establishing protection from pathogens through primary immunity [5], [6]. DC are required for the induction of antigen specific immune responses including cell proliferation and functional maturation of specific T cell clones [6], [7].

Based on pioneering work in animals and humans [8], [9], [10], promising studies in late stage cancer patients [11], [12], [13], and our own pre-clinical activities [14], we completed a pilot study in healthy, low-risk, HIV uninfected volunteers to compare direct *ex vivo* targeting of DC with standard immunization routes. We reasoned that DC vaccination would enhance the immune response and improve our understanding of optimal immunization. We used monocyte derived DC to generate large numbers of cells for vaccination similar to those used in other DC vaccination protocols [15]. Monocytes were recently shown to fully differentiate *in vivo* into potent antigen presenting DC-SIGN expressing DC that localize to the lymph nodes after appropriate stimulation [16].

We selected an experimental canarypox vaccine previously tested in adults and children, vCP205, containing HIV-1 *env*, *gag* and *pol* genes. This particular study design was novel as autologous DC vaccination had been limited to therapeutic studies, and not yet used in healthy donors for a preventative vaccine. Our results show that multiple autologous *ex vivo* loaded mature DC vaccinations were well tolerated and more immunogenic than conventional vaccine delivery routes.

## Materials and Methods

### Objectives

The study objectives were to assess the safety, tolerability and immunogenicity of ALVAC-HIV administered subcutaneously via *ex vivo* transfected autologous DC, intradermally, or intramuscularly.

### Ethics statement and regulatory approval

The clinical protocol was entitled “A Phase I Study of Aventis Pasteur Live Recombinant ALVAC-HIV (vCP205, HIV-1 env/gag/pol) in Seronegative Adults Administered (1) SQ via *ex vivo* Transfected Autologous Dendritic Cells, (2) Intradermally, or (3) Intramuscularly”. This protocol, designated RV138 by nominal convention within our program, was sponsored by the Office of the Surgeon General and approved by the Institutional Review Board of the Division of Human Subjects Protection, Walter Reed Army Institute of Research. This study was conducted under the auspices of the US FDA, IND #BB12207, and registered with the NIH clinical trials.gov as NCT#00013572. All volunteers provided written informed consent following discussion and counseling by the clinical study team prior to enrollment and before any study related procedures were performed.

### Study design

Eligible participants were randomized to one of three delivery routes: Arm 1: SQ via *ex vivo* loading of autologous DC, Arm 2: direct ID injection or Arm 3: direct IM injection. We used a rolling enrollment process and allowed replacements of volunteers while the study was open to reach a target total n = 36 individuals, 12 per group. Within each group, volunteers were further randomized to receive either vCP205 vaccine or placebo at a 2∶1 ratio. Vaccinations were given at 0, 1, 3 and 6 months. All subjects underwent leukapheresis pre-vaccination and 2 weeks post-vaccination. Other routine blood draws were performed per protocol throughout the study. All volunteers were observed for one hour post-vaccination, contacted by telephone within 48 hours post-vaccination and returned to the clinic for a 2-week post-vaccination safety visit. Routine safety visits included solicitation of adverse events in the interval since their last visit, review of a 7-day diary card, directed physical exam and routine safety labs. The trial protocol and CONSORT checklist can be found in the supporting information; see Checklist S1 and Protocol S1.

### Participants and setting

RV138 was conducted in Rockville, MD within the Military HIV Research Program where all samples and data were collected. Eligible volunteers were healthy, aged 18–55 years, HIV uninfected and low risk for acquisition of HIV infection (e.g. no intravenous drug use, no known sexual exposure to HIV+ partner). Female participants agreed to avoid pregnancy for the duration of the study and a negative serum pregnancy test was required prior to each vaccination. Full details of inclusion and exclusion criteria may be found in the protocol (Protocol S1).

### Interventions

ALVAC-HIV (vCP205, Aventis Pasteur, France) is a recombinant canarypox virus expressing HIV-1 *env*, *gag* and *pro* subtype B gene products. The vCP205 env is comprised of a gp120 MN + a TM gp41 LAI. The HIV genes are inserted into the C3 locus and are regulated by the vaccinia virus H6 and I3L promoters. The lyophilized vCP205 vaccine (10^6.5^ TCID_50_/vial) was reconstituted to 1 ml and administered at the following doses: 1.0 ml IM into deltoid, 0.5 ml ID given by 4×0.125 ml injections into the volar aspect of forearm, and approximately 3–6×10^6^ DC were injected in the superficial subcutaneous aspect of the inner arm within 10 cm of the axilla. For Groups II and III, saline was administered as the placebo control. For the DC arm (Group I), those receiving placebo were administered autologous DC pulsed with KLH (injection 1) or no antigen (subsequent injections).

### Outcomes

The primary endpoints were the evaluation of safety and tolerability as well as immunogenicity. All subjects were observed in the clinic for local and systemic reactions post vaccination and instructed to report any reactions occurring over the next 7 days. Routine safety labs were performed 14 days after each vaccination, along with a physical examination. Primary measures of immunogenicity included lymphocyte proliferation, CTL responses and IFN-γ production in response to vaccine component antigens. Ancillary immunogenicity measures included assessment of the breadth of humoral responses to vaccine component antigens, mapping of cross-reactive cellular epitopes and analysis of the cytokine profile produced during lymphocyte proliferation.

### Sample Size

This study was intended as an exploratory proof-of-concept to test the safety of two new routes of vaccine delivery via dendritic cells and intradermal injection. A sample size of 12 volunteers per group (8 active product and 4 placebo) provided approximately 70% power to detect a true difference, should one exist, in CTL response between the DC arm and the better of the IM and ID arms. All subjects receiving at least one injection of vaccine were included in the safety and tolerability analyses.

### Randomization and blinding

Subjects who met initial admission criteria were assigned personal identification numbers and screening numbers at the first screening visit. After a volunteer was deemed eligible, the screening number became the study ID number. All three arms were enrolled concurrently and subjects were randomly assigned to an arm. Within each arm subjects were randomly assigned to active vaccine or placebo according to a list generated by the MHRP Data Coordinating and Analysis Center. Study site staff and volunteers remained blinded with respect to the allocation of placebo or vaccine, but not to study arm.

### Cells

Peripheral blood mononuclear cells (PBMC) were isolated from apheresis products obtained at visits 1 (v.1) and 11 (v.11), or from whole blood drawn at alternate visits. Apheresis product or whole blood was diluted with PBS, layered over Ficoll-Paque Plus (GE Healthcare, Piscataway NJ), centrifuged and harvested as previously described [14]. The apheresis products were washed more extensively than whole blood derived PBMC with large volumes of PBS to remove platelets. Cells were cryopreserved in 10% dimethyl sulfoxide (Sigma, St. Louis MO), 12.5% human serum albumin (SeraCare Life Sciences, West Bridgewater MA), X-VIVO-15 media (BioWhittaker/Lonza, Walkersville MD) and maintained in vapor phase liquid nitrogen storage until use.

### Generation of clinical-grade DC

Autologous clinical-grade DC were generated as described [14], [17]. Briefly, monocytes isolated by adherence to tissue culture dishes were incubated for 6 days in RPMI-1640, 1% autologous heat inactivated human plasma, 2 mM L-glutamine (BioWhittaker/Lonza) and 20 mg/ml gentamicin (American Pharmaceutical Partners, Los Angeles CA) supplemented with the recombinant cytokines IL-4 (800 U/ml; CellGenix, Antioch IL) and granulocyte-macrophage colony-stimulating factor (GM-CSF, 1000 U/ml; McKessen, Richmond VA). To generate mature DC, 20% vol/vol autologous monocyte conditioned media (MCM) was added on day 6 and cells were harvested on day 7 [14]. DC phenotype and maturation was assessed by flow cytometry using phycoerythrin (PE)-conjugated monoclonal antibodies to CD1a, CD3, CD14, CD20, CD25, CD80, CD86 and HLA-DR (BD Biosciences, San Jose CA) and CD40 and CD83 (Beckman Coulter, Fullerton CA). The pre-specified release criteria included maturation (mDC ≥50% CD86^+^ and/or CD83^+^), >70% viability, with a negative sterility panel including gram stain, aerobic and anaerobic cultures, mycoplasma culture and PCR, and endotoxin (Limulus amoebocyte lysate assay).

### *Ex vivo* loading of DC with vaccine

DC vaccinations were prepared fresh for every injection. For the first 14 vaccinations in 4 donors, immature DC were first matured overnight with MCM and then loaded with vaccine the following morning. However, the loading sequence was modified during the trial in order to increase intracellular p24 expression, as an estimate of vaccine-loading efficiency. For all subsequent vaccinations, the DC were first loaded with vaccine in the immature state, followed by overnight MCM maturation prior to injection. For the first vaccination in all donors, their DC were exposed to keyhole limpet hemocyanin (KLH, Calbiochem/EMD, Gibbstown NJ or Biosyn, Lewisville TX)) as a control for immune response to a neoantigen and DC integrity. The maximum number of available DC, up to 6×10^6^, were injected at each vaccination.

### Measurement of serum IgG titers by ELISA

The following antigens were coated overnight at 4°C onto Immulon-2 microtiter plates (Fisher Scientific, Pittsburgh, PA): p24 (0.5 µg/ml, ImmunoDiagnostics, Inc.); gp160-MN/LAI (0.5 µg/ml, PMC, France); and ALVAC CPpp (4 µg/ml, Aventis Pasteur, France). The p24 and gp160-MN/LAI were coated in PBS, pH 7.4 containing 0.01% thimerosal. The ALVAC CPpp was coated onto plates in bicarbonate buffer (dH_2_O, sodium carbonate monohydrate, sodium bicarbonate, pH 9.6). Plates were blocked with serum diluent for 1 hour prior to serum addition then washed with buffer (PBS with 0.1% Tween 20, pH 7.4). Serum (100 µl in two-fold dilutions in wash buffer with 5% skim milk, pH 7.4) was added for 1 hour at 37°C. After incubation with the sera, plates were washed and 100 µL of horseradish peroxidase-conjugated goat anti-human IgG was added (Kirkgaard & Perry, Gaithersburg MD) to each well for 1 hour at 37°C. Plates were washed, TMB substrate (Kirkgaard & Perry) was added for 15 min and the reaction was stopped with 1 M phosphoric acid. The optical density for each well was read at a wavelength of 410 nM on a VersaMax reader (Molecular Devices, Inc., Gaithersburg MD).

### Cytotoxic T lymphocyte (CTL) assay

A standard chromium release CTL assay was performed on freshly isolated PBMC with in 6 hours of collection as previously described [18]. *In vitro* stimulations were set up using 20×10^6^ freshly isolated PBMC, of which 20% (stimulators) were infected with a vaccinia recombinant expressing HIV subtype B *env* gp120 MN gene and B gag/pol IIIB genes (vP1291) for 14±2 days. Autologous EBV-transformed B cells (TBC) were infected overnight with vaccinia recombinants (Virogenetics, Troy NY) expressing subtype B (MN) *env* (vP1174) and subtype B (IIIB) *gag/pol* (vP1287), or empty vector (vP1170), and labeled with ^51^Cr for use as targets in the CTL assay. Cold target empty vector infected TBC were used to compete and absorb background vaccinia responses. CTL assays were performed at effector to target (E∶T) ratios of 50∶1 and 25∶1, with whole, CD8- or CD4-depleted PBMC in order to verify CD8 restriction. Specific lysis was calculated as [(experimental release-spontaneous release)/(maximum release-spontaneous release)]×100. Background vaccinia responses were subtracted from HIV-1 specific responses. A positive CTL is defined as specific lysis greater than 10% at either E∶T ratio, provided that elevated lysis at 25∶1 is supported by lysis at 50∶1. The response was CD8^+^ T cell mediated if depletion of CD8^+^ T cells removed 50% of the HIV antigen specific lytic activity, while removal of CD4^+^ cells maintain at least 5% HIV specific lysis.

### IFN-γ ELISPOT assay

The ELISPOT assay, as described previously [18], was performed at visit 1 and 9 additional visits throughout the trial. Fresh PBMC (1–2×10^5^) were added in duplicate wells, or for the PBMC only wells in quadruplicate. Pools of peptides matched to the vaccine strain (see below) or controls were added at a final concentration of 2 µg/ml. Determination of CD8^+^ T cell dependence was achieved by immunomagnetic bead depletion (Dynal, Upsalla, NY). The ELISPOT plates were examined under a stereomicroscope and spots were evaluated with an Automated Elispot Reader System with KS 4.3 software (Carl Zeiss, Thornwood, NY). The results are expressed as the number of IFN-γ secreting cells or spot forming cells (SFC) per million PBMC. A positive response was defined as >4× background and >55 SFC/10∧6 PBMC, based on the Merck Laboratory definition [19].

### Synthetic HIV peptides and other reagents

Peptides were obtained from the NIH AIDS Research and Reference Reagent Program, Division of AIDS, NIAID, NIH. Peptides (15mers overlapping by 11, with a minimum purity of ∼80%) were pooled for use in the ELISPOT assay; 212 env (catalog #6451) and 122 Gag (catalog #5107) peptides were made into 9 and 5 pools of 25 peptides, respectively. A pool of 23 peptides synthesized in-house using Fmoc chemistry and standard solid-phase techniques (Excel automated synthesizer; Waters, Milford MA) consisting of MHC class I epitopes from CMV, EBV and Flu (CEF pool, [20]) was used as a positive control.

### Lymphocyte proliferation assay (LPA)

PBMC were plated at 1.5×10^5^ cells/well in 96-well plates in media alone or with antigen in triplicate wells and incubated for 6 days at 37°C, 5% CO_2_. Antigens included aldrithiol-2 inactivated HIV-1 MN (AT-2 HIV, 100 ng capsid antigen/ml, lot #3763 courtesy J. Lifson, AIDS and Cancer Virus Program, SAIC-Frederick, Inc., NCI Frederick, Frederick MD); KLH, 100 µg/ml; HIV-1 gp160 (10 µg/ml) and HIV-1 p24 (12.5 µg/ml), both from Advanced BioScience Laboratories, (Kensington, MD). Microvesicles (MV, Jeff Lifson, NCI) from uninfected cultures were used at an equivalent protein concentration as a negative control for AT-2 HIV, and phytohemagglutinin (1 µg/ml, Sigma, St.Louis, MO) was used as a positive control. The cells were pulsed with 1 µCi/well [^3^H]thymidine and harvested onto filter mats for scintillation counting on day 6. Small volumes of cell free supernatants were pooled from replicate wells at specified intervals for cytokine analysis.

### Fluorescent labeling of PBMC

Thawed PBMC were labeled with carboxy-fluorescein dicetate succinimidyl ester (CFDA-SE) according to manufacturer's instructions (Invitrogen, Carlsbad CA). Briefly, cells were labeled with 2.5 µM CFDA-SE in PBS supplemented with 5% fetal calf serum at 37°C. To quench the reaction, cells were washed in cold 5% FCS/PBS and washed twice with proliferation media. Cells were plated at 2×10^6^ cells/well in 48-well plates in media alone or with antigens as used in the LPA. Following a 6-day incubation cells were harvested and stained for surface marker expression using monoclonal antibodies against CD3 (PE conjugate), CD4 (allophycocyanin (APC) conjugate) and CD8 (peridinin chlorophyll protein (PerCP) conjugate) (BD Biosciences).

### Quantitation of cytokines

Supernatants from LPAs at days 2 and 6 were quantified using human cytometric bead array kits (BD Biosciences) per manufacturer's protocol. Cocktails of capture beads for IL-1β, IL-2, IL-4, IL-6, IL-10, IL-12, INF-γ, or TNFα were added to a 96-well filter-plate (Millipore) with 50 µL supernatant. After 1 h, 50 µL PE detection reagent was added to each well and the plate was incubated for 2 hours. Samples were washed and resuspended in 150 µL wash buffer, collected on an LSR-II flow cytometer and analyzed with FCAP Array software (BD Biosciences).

### Gene regulation assessment

Total RNA was extracted from PBMC stimulated with either AT-2 HIV or control MV, assessed for quality and quantity and prepared for hybridization to the Affymetrix Human Genome Focus Genechip essentially as described previously [21]. Data were normalized using Robust Multichip Averaging. All nonexpresseed genes were eliminated from further analysis. Differentially expressed genes were identified using Significance Analysis of Microarrays (SAM) v1.21 and the Database for Annotation, Visualization and Integrated Discovery.

### Statistical analysis

All assays used preset criteria for positivity. For CTL and ELISPOT assays, the following criteria were also applied 1) the presence of repeat responses over multiple time points; more than one positive response post-vaccination and 2) data for individuals where the baseline samples were positive were not included in the analysis. Statistical analyses for CTL and ELISOPT were performed independently by the Data Analysis and Coordinating Center at MHRP. Additional statistical analyses were performed using Prism (GraphPad Software, LaJolla CA).

## Results

### Enrollment, participant flow and demographics

**Figure 1:**
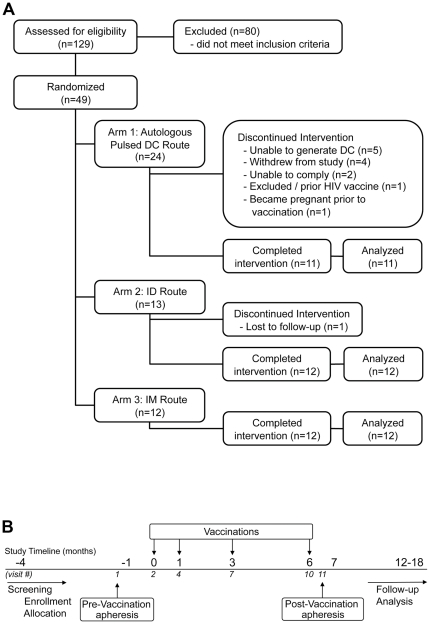
Study Design. (A) Consort diagram. (B) Study timeline. Vaccinations were administered at 0, 1, 3 and 6 months. All study subjects underwent apheresis prior to initial vaccination (visit 1) and 2 weeks following the final vaccination (visit 11).

Forty-nine healthy HIV seronegative volunteers were randomized to the three study arms: (1) autologous pulsed DC (n = 24), (2) ID injection (n = 13) or (3) IM injection (n = 12) (Consort diagram, Fig. 1a). Two weeks prior to the first vaccination (v.1), all subjects underwent apheresis to obtain PBMC for the generation of autologous DC (DC arm only) and for baseline *in vitro* immunologic measurements (all arms) (Fig.1b). We were unable to generate DC from 5 of the volunteers due to a technical issue with a manufactured reagent. One volunteer became pregnant prior to vaccination, a second was excluded due to prior receipt of an HIV vaccine and a total of 7 exited the study prior to vaccination. The last donor randomized to the DC arm was later excluded after enrollment closed and was not replaced, This resulted in only 11 total volunteers in this arm. All 35 volunteers who completed their vaccination series (ID, IM or DC) were included in the immune analysis. The median age of subjects was 42.2 years with an even distribution of males and females in each group. As previously reported [22], our study demographics reflect the greater Washington, DC region, see Table 1. Vaccinations were given at 0, 1, 3 and 6 months. A post-vaccination apheresis was performed 2 weeks after the final vaccination (v.11) for presumed “peak immunogenicity”.

**Table 1:** Demographics of study volunteers.

Study Arm	N	Median Age	Male∶Female	Racial Demographics				
			Ratio	African Amer.	Caucasian	Hispanic	Asian	Other
DC	11	42.3	6∶5	36%	55%	9%	0%	0%
ID	12	41.6	6∶6	42%	50%	0%	8%	0%
IM	12	41.8	5∶7	33%	58%	0%	0%	8%
**Total**	**35**	**42.2**	**17∶18**	**37%**	**54%**	**3%**	**3%**	**3%**

### Safety and tolerability

**Figure 2:**
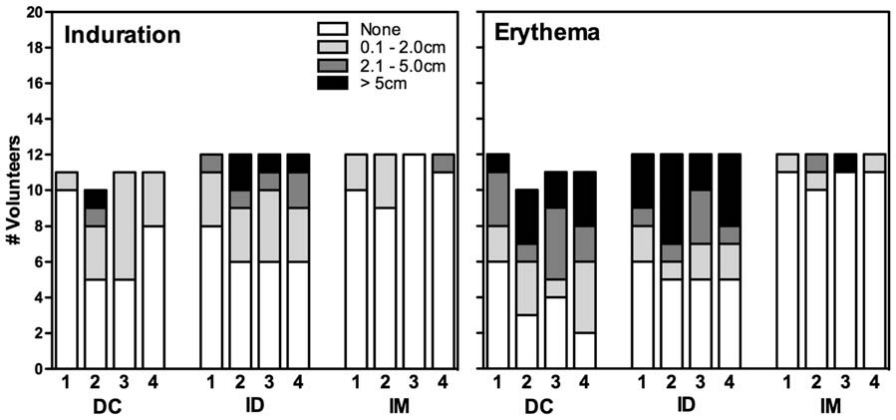
Post-vaccination reactogenicity. Post injection-site reactogenicity symptoms including induration (left panel) and erythema (right panel) after each vaccination for all study groups (autologous DC (DC), intradermal (ID) and intramuscular (IM) routes).

All vaccinations and delivery routes were well tolerated. After each vaccination, volunteers were observed in the clinic for 60 minutes for signs of local or systemic reactions. They were educated on how to use a diary card to record their symptoms for the week after vaccination. Diary cards were reviewed and volunteers were assessed for symptoms at the subsequent safety visit. The post vaccination reactions were mild, transient, mainly local and typically included erythema, induration (Fig. 2), tenderness or warmth (data not shown). The highest frequency of local reactions was in the ID group, with fewer in the DC group and very few in the IM group. Some subjects reported mild systemic events including headache or transient myalgia, but most events did not require analgesics, nor did they interfere with the subjects' daily routine. No severe adverse events occurred.

### Humoral responses

**Figure 3:**
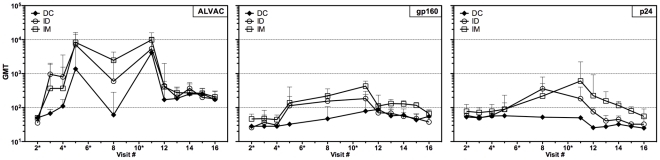
Antibodies develop after vaccination. Humoral responses to the vaccine vector (ALVAC, left panel) and insert genes HIV-gp160 (middle panel) and HIV-p24 (right panel). Data points are the geometric mean titers (GMT)+SEM bars for all subjects within each study arm receiving vaccine. Visit numbers are indicated and an asterisk (*) denotes vaccination visits. Closed diamonds, autologous DC route; open circles, ID route; open squares, IM route.

Antibody responses to the ALVAC vector and the HIV proteins env gp160 and gag p24 were monitored throughout the study by ELISA. Geometric mean binding antibody titers (GMT) detected over a two-year period for all tested antigens are shown (Fig. 3). As expected, after the final vaccination, the canarypox vector elicited strong responses (v.11, >10,000 GMT) in all groups. This response tapered off yet remained positive (v.16, >200 GMT) for 18 months post vaccination. The gp160 responses were highest in the IM route and comparable in the ID and DC routes. All gp160 responses decreased over time and reverted to negative 18 months after vaccination (v.16, <100 GMT). The ELISA gag responses were lowest in magnitude and especially notable for their absence in the DC arm.

### CTL and ELISPOT responses

IFN-γ ELISPOT responses were assessed from fresh PBMC processed within 4 hours of blood draw using vaccine-matched peptides. Two of seven individuals in the DC arm had HIV-gag IFN-γ ELISPOT responses and 1/8 individuals in the IM arm had HIV-env IFN-γ ELISPOT responses. In these individuals, the responses were seen at more than one time point post vaccination, were of modest magnitude and were CD8 restricted as defined by magnetic bead depletion studies. HIV-env CD8 specific CTL responses as measured by ^51^Cr release were detected in 2/8 individuals in the IM arm (Table 2). Since one of the IM participants had responses detected over multiple time points by ELISPOT, CTL and whole blood intracellular cytokine staining (ICS, data not shown), we further explored this individual's immune response at v.16, collected 18 months after the last vaccination. At this visit, fresh CTL lysis was 70% and 50% at E∶T of 50∶1 and 25∶1 respectively, and confirmed to be CD8 T cell dependent. The *in vitro* CTL culture was maintained and expanded using IL-2 and autologous B cells expressing MN Env. A matrix ELISPOT with the *in vitro* expanded PBMC was performed and an Env epitope CTRPNYNKRKRIHIG (B0703) was mapped in this individual with frequencies of 484 and 979 SFC/10,000 PBMC. The *in vitro* expanded PBMC were phenotyped in an ICS and further tested for CTL recognition of Env CTRPNYNKRKRIHIG peptide and vaccinia MN pulsed B cell targets. The phenotype of the cells was confirmed to be CD8 positive (41.5% CD3^+^/CD8^+^) and these cells mediated 70% killing of peptide and vaccinia MN pulsed B cell targets at an E∶T ratio of 20∶1 as shown in Figure S1.

**Table 2:** CTL and IFN-γ ELISPOT responses to any HIV antigen.

	IFN-γ ELISPOT		CTL	
Arm	Placebo	Vaccine	Placebo	Vaccine
ID	0/4	0/8	0/4	0/8
DC	0/4	2/7^a^	0/4	0/7
IM	0/4	1/8^b^	0/4	2/8
Total # responders	0/12	3/23	0/12	2/23
Positive control responses	12/12^c^	23/23^c^	na	na

a *gag responses,*

b *env responses,*

c *PHA responses.*

### HIV specific lymphoproliferative responses

**Figure 4:**
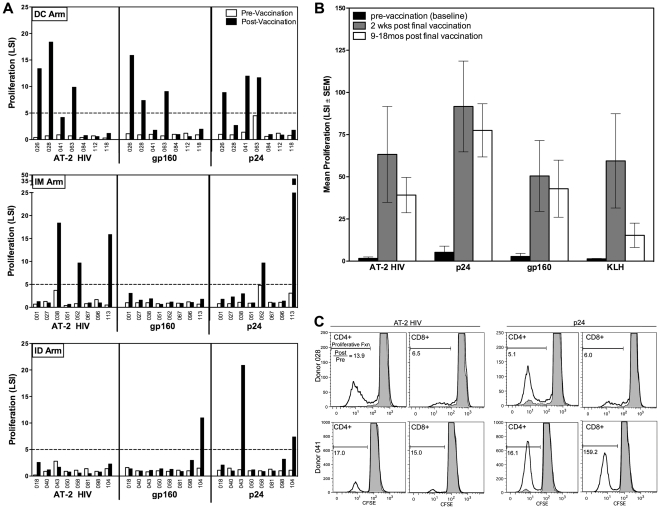
HIV specific lymphocyte proliferation responses. LPA responses of PBMC from (A) pre-vaccination (v.1, white bars) and two weeks post final vaccination (v.11, black bars) from all vaccinees. Responses to whole inactivated HIV (AT-2 HIV), HIV-gp160 and HIV-p24 were measured and reported as a lymphocyte stimulation index (LSI, antigen response/unstimulated control). An LSI of >5 (dashed line) is considered positive. (B) Durability of proliferative responses up to 18 months post final vaccination for the 4 responders in the DC vaccination arm. Responses to HIV antigens, keyhole limpet hemocyanin (KLH, positive control for DC injection) and aldrithiol-2 microvessicles (MV, negative control for AT-2 HIV) are expressed as mean LSI±SEM from LPAs performed on fresh PBMC. (C) Proliferative responses pre-vaccination (grey histograms) and 2 weeks post final vaccination (black lines) to HIV antigens were measured by flow cytometry using CFDA-SE. CD4^+^ and CD8^+^ T cell responses are shown for two donors from the DC arm stimulated with either AT-2 HIV (left panels) or HIV-p24 (right panels). Proliferative response is measured as the percent of cells that have undergone division and show reduced intensity of CFSE. The ratio of proliferative response post-vaccination to pre-vaccination is shown.

Pre- and post-vaccination PBMC were assayed for HIV specific responses in the LPA (Fig. 4A). In the DC arm, 4/7 vaccinees responded to at least one of the HIV antigens tested, with 3/7 responding to all three antigens tested. Responses in the IM group were seen to AT-2 HIV and p24, but not to gp160. The ID arm showed the most limited responses: 2/8 total responders, both responsive to p24, one responsive to gp160, and none to AT-2 HIV. We further examined whether the responses seen at 2-weeks post-vaccination were durable. PBMC from all four DC arm responders, isolated from apheresis or whole blood draws over the time period covering pre-vaccination up to 18 months post-vaccination, were tested. After the third vaccination, cells remained strongly responsive to the antigens, but not the negative control, with at least 18-month durability after the final vaccination (Fig. 4B).

### Identification of proliferating cell populations

Along with the traditional ^3^H-thymidine incorporation LPA assay, cells were subjected to identical antigen stimulation conditions in a flow cytometry-based proliferation assay. PBMC were labeled with CFDA-SE prior to stimulation with antigen. Co-labeling with monoclonal antibodies to CD3, CD4 and CD8 allowed for the identification of dividing T cell populations. Figure 4E shows overlays of pre-vaccination and post-vaccination CD4^+^ and CD8^+^ populations of two representative DC arm responders. While the CD4 T cells are the predominant dividing population, there is evidence of proliferation within the CD8 T cell compartment in response to both AT-2 HIV (left panels) and to p24 (right panels).

### Effect of antigen loading sequence in the DC arm

**Figure 5:**
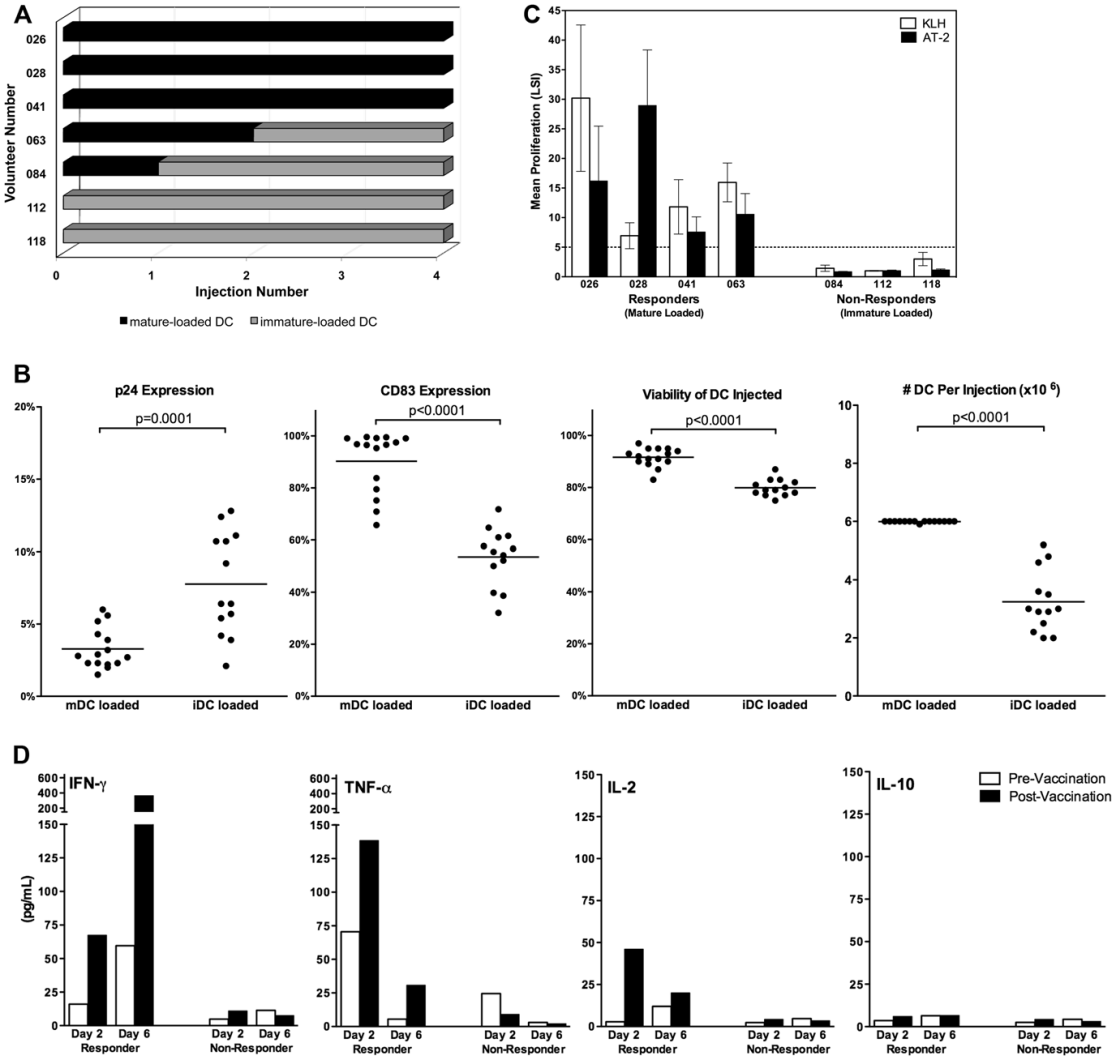
DC vaccine characterization. (A) Schematic representation of the DC maturation sequence. Autologous DC were loaded with vaccine either after maturation (black bars) or before maturation (grey bars). (B) Characteristics of vaccine-loaded DC, segregated by loading/maturation sequence. Expression of HIV-p24 and the maturation marker CD83 were assessed by flow cytometry. The number of DC per injection and cell viability at the time of injection were assessed by visual counts using Trypan Blue exclusion. P-values represent the statistical difference as determined by an unpaired t-test. (C) Proliferative responses of DC arm vaccinees to KLH (white bars) and AT-2 HIV (black bars). Data is shown as mean LSI±SEM from LPAs performed on fresh PBMC isolated between visits 1–16. (D) Cytokine secretion following AT-2 HIV stimulation of PBMC collected pre-vaccination (v.1, white bars) and two weeks following the final vaccination (v.11, black bars). IFN-γ, TNF-α, IL-2 and IL-10 secretion from representative responder and non-responder vaccinees from the DC arm are shown.

Pre-clinical experiments suggested that maturing the DC before loading them with vaccine yielded a population of cells that were fully mature and expressing p24 [14]. Midway through the study, we compiled the mean p24 expression in the injected DC populations and found it was below the predetermined minimum of 5% expression. To improve p24 expression, the loading sequence was inverted so that immature DC were loaded with vaccine and subsequently matured. Figure 5A shows the distribution of DC vaccine injections based on loading sequence. Three patients received only mature-loaded DC, two patients received only immature-loaded DC, and two received a combination. DC vaccinations were characterized and compared based on loading sequence for p24 (loading efficiency), CD83 expression (maturation level), viability and number DC per injection (Fig. 5B). Overall, the immature-loaded DC expressed statistically higher levels of p24 (2-fold more) than the mature-loaded cells. Following maturation of the immature-loaded cells, significant decreases were seen in expression of CD83 and cell viability. Decreased viability, along with other technical issues in recovery of the immature-loaded DC, lead to fewer total DC available for injection (6 M vs. ≥3 M). The decrease in responses in the DC arm vaccinees to the control protein KLH, as well as AT-2 HIV, coincided with the change in DC loading sequence (Fig. 5C).

### Cytokine production

We measured cytokines in the LPA supernatants at days 2 and 6 using human cytometric bead array assays for IL-1β, IL-2, IL-4, IL-6, IL-10, IL-12, INF-γ, and TNFα. We focused on IFN-γ, TNF-α, IL-2 and IL-10 production at pre- and post-vaccination time points (v.1 and v.11, respectively, Fig. 5D) because they are commonly monitored in vaccine studies (IFN-γ, TNF-α and IL-2) and relevant with regard to “tolerance” or “ignorance” in the case of IL-10. Representative data from DC arm responder and non-responder subsets are shown. Early IL-2 and TNF-α were detected followed by late IFN-γ in the responders, while minimal cytokine production was observed in the non-responders. IL-10 was not detected in any donor supernatants. Because prior reports indicated that immature antigen-loaded DC could tolerize the immune response [8], we conducted flow cytometric analysis of pre- and post-vaccination PBMC from 10/11 of the DC vaccinees, responders and non-responders, using FoxP3, CD27, CD25 staining. There were no increases in T_reg_ cells observed in any DC vaccine recipient throughout the study (data not shown).

### Genomic analysis

**Figure 6:**
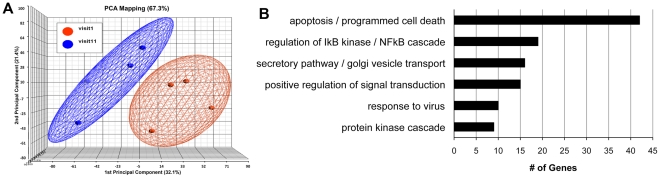
Antigen-specific differential gene expression. (A) Principal components analysis of differentially expressed genes for the group of responders in the DC arm. Red spheres denote pre-vaccination state (Visit 1) and blue spheres denote post-vaccination status (Visit 11). The X-axis is the first principal component, the Y-axis is the second principal component and the Z-axis is the third principal component. (B) Functional groups of gene families most significantly down-regulated in response to *in vitro* AT-2 HIV stimulation of PMBC from the DC arm responders.

Following *in vitro* stimulation with AT-2 HIV, RNA extracted from pre- and post-vaccination PBMC from the 4 DC arm responders was evaluated by gene array analysis for differential gene expression. After stimulation with AT-2 HIV, but not with control MV or media alone, distinct gene expression patterns emerged between the pre-vaccination and post-vaccination samples (Fig. 6A). The patterns were statistically significant and resolvable in a stringent analysis using a false discovery rate of <1%. Correlating with the proliferation and cytokine responses, there was no differential expression seen in the pre-vaccination cells, nor in any of the post-vaccination cells of the non-responder subset. However, in the post-vaccination cells in the responder subset, 2 genes were upregulated and 203 down regulated (Table 3). The functional groups most affected during the proliferation responses included those controlling apoptosis and programmed cell death, response to virus, and regulation of NFκB (Fig. 6B).

**Table 3:** Differential gene expression after *in vitro* stimulation with whole-inactivated HIV.

	# of genes up-regulated		# of genes down-regulated	
Stimulus	NR^a^	R^b^	NR	R
media alone	0	0	0	0
control microvesicles	0	0	0	0
AT-2 HIV	0	2^c^	0	203

a *Non-Responders,*

b *Responders,*

c *oligophrenin-1 and neuropeptide ff receptor-1.*

## Discussion

Classical vaccination techniques, centered on the induction of humoral and cellular immunity against HIV, have yielded disappointing results. However, recent data from the Phase III, RV144 Thai study using a similar canarypox viral vector (vCP1521) and a protein subunit boost showed a modest (31%) reduction in HIV acquistion despite minimal T cell responses as detected by IFN-γ ELISPOT [4]. This level of ELISPOT activity, in the range of 15–40%, is fairly typical for ALVAC-HIV studies [23], [24], [25]. Notably, most vaccinees in RV144 showed lymphoproliferative responses, CD4 responses to HIV-env by ICS and developed binding antibodies to vaccine components. The only difference between the canarypox vector in this study (vCP205) and the Thai trial (vCP1521) is in their respective env sequences: here in RV138 we used an MN (subtype B) and in the RV144 Thai trial, 92TH023 (subtype A/E) was used. Immune correlates of protection are under active investigation for the Thai study. In this context, it is incumbent on researchers to explore new avenues for vaccine development and broaden our understanding of the assessment of immune responses to vaccination. To this end, we conducted a randomized, controlled double-blinded HIV vaccine trial comparing standard immunization routes to a novel delivery route using autologous *ex vivo* loaded dendritic cells. This DC targeted approach to vaccination is labor intensive and ultimately non-deployable on a large scale, but can be informative for vaccine development.

We found that all administration routes of vCP205, including multiple rounds of autologous DC vaccination of healthy volunteers, were safe and well tolerated. Similar to the RV144 Thai study, our study generated predominantly proliferative responses in the CD4 T cell compartment. Qualitative differences were detected in immune responses that were route dependent with the *ex vivo* targeted DC being more immunogenic *>* IM > ID as assessed in the functional LPA. Somewhat surprisingly, the ID route, which targets a DC rich zone and is proposed to enhance vaccine efficacy (22), induced the most limited of cellular responses. One possible explanation is that we used a lower dose of ID vaccine (50%) given the volume constraints of the dermal compartment. We hypothesized that a reasonable dose-sparing effect should be observable with half the dose. This pilot study suggests there was no observed dose sparing effect giving the vaccine intradermally.

There were clear responders and non-responders to DC vaccination. In fact, the first 4 consecutive volunteers responded to DC vaccination, whereas the last three did not. The presence or absence of an HIV response coincided with the KLH responses (Fig. 5C). Therefore, KLH served as an important control neoantigen, indicating loss of DC integrity or compromise after loading with a live viral vaccine since both active vaccinees and placebo recipients' DC were pulsed with KLH. It is important to note that KLH specific CD8 T cell ELISPOT responses were low in the DC arm (data not shown). The lymphoproliferative responses elicited by DC vaccination were broad. Responses were detected to whole inactivated HIV-1, as well as env (gp160) and gag (p24) proteins. These responses were robust and durable; lasting up to 18 months post vaccination (Fig. 4B). In an effort to better characterize the responding cells, we used the cell tracking dye CFDA-SE along with phenotypic markers in a flow cytometric assay. While the preponderance of dividing cells were CD4^+^ T cells, there was detectable proliferation in the CD8^+^ compartment (Fig. 4C). Despite proliferative activity in the CD8 T cell compartment, cytolytic activity was low. This could be consistent with a recent report that suggests that in the murine cancer setting, priming of a CD8 immune response is indirectly mediated by DC [31].

A key difference between responders and non-responders was their DC maturation status at the time of vaccine loading. Maturing the DC first, then loading the vaccine, resulted in greater immune responses despite significantly reduced p24 expression (Fig. 5B, left panel). It is possible that the mature loaded DC were more functional and capable of priming immune responses to the vaccine. When the DC were loaded with vaccine in the immature state, though they expressed more p24, there was reduced viability, decreased maturation (CD83 expression) and technical difficulties which lowered the overall cell yield for vaccination. In addition to our experience, independent reports implicate a role for type I interferons in ALVAC loaded DC showing that ALVAC exposed monocyte derived DC upregulate the type I interferon signaling pathway and DC maturation status influences downstream intracellular networks via a “molecular switch” in IFNα/β signaling [32], [33]. Taken together, integrating our work and the existing literature, we suggest that mature DC are better able to withstand viral vaccine loading and function as primary inducers of immune responses. Our poxvirus data is consistent with prior reports indicating immunologic benefit when loading mature DC with either peptides or RNA [34], [35].

As prior publications suggested that antigen loaded immature DC were tolerogenic, we looked for, but did not find, either HIV specific IL-10 production or the induction of T_reg_ cells after vaccination [36], [37]. It is unclear whether DC non-responders were unresponsive in an antigen specific manner or simply ignored the signal having been insufficiently primed at the outset. The absence of IL-10 (Fig. 5D) and stable circulating T_reg_ phenotypes and frequency pre- and post- vaccination (data not shown), are reassuring observations, leaving open the idea that optimal DC maturation and targeting could lead to more potent vaccines. Data from our gene expression experiments also supports the idea that cells from the non-responders were likely unresponsive, as opposed to tolerized. This assay was designed as an adjunct to our functional studies. We aimed to detect differences in gene regulation between responders and non-responders when stimulated with HIV antigens under LPA like conditions. The data show significant differences between the two groups. In response to stimulation with AT-2 HIV, post-vaccination cells from the responders showed differential regulation of 205 genes. In contrast, no effect was seen in the non-responder group, or in any of the pre-vaccination samples (data not shown).

Cytolytic CD8 T cell response rates, particularly ELISPOTs and chromium release CTLs, were low overall and similar to those reported in previous canarypox studies [4], [23], [24]. One of the few responders to generate a strong CD8 cytolytic response was vaccinated by the IM route. This vaccinee responded to a peptide sequence CTRPNYNKRKRIHIG, corresponding to position 296–301 (HXB2 numbering) in the V3 loop of the envelope protein. This HLA-B0702 restricted peptide sequence appears to be highly specific to MN subtypes and represents the first time vaccine induced immune responses have been reported against this epitope. While similar sequences denoted B7-R110 have been reported in acute [26] and chronic [27], [28], [29] HIV-1 infection, there is no known protection associate with this epitope [30].

Pilot studies are helpful in finding early safety signals, generating hypotheses and driving innovation. Multiple doses of HIV-1 vaccine pulsed, *ex vivo* loaded autologous DC were safe and well tolerated in normal healthy people and immunogenic *in vivo*. Study limitations include the small study size, multiple arms and the change in DC arm loading sequence that was not part of the original study design. Additionally, we did not inject ALVAC SQ as a comparator to the *ex vivo* ALVAC loaded DC injected subcutaneously. Subset analysis of the DC responders and non-responders were included for information purposes but cannot be considered highly robust datasets. Yet, antigen specific proliferative immune responses were observed in both CD4^+^ and CD8^+^ T cell compartments after DC vaccination and were associated with IL-2, TNF-α and IFN-γ production. Though a few DC vaccinees did not respond to the vaccination series, no evidence for T_reg_ cell induction or tolerance was found. Non-responders and responders were readily differentiated using expression profile analysis supporting our results, but given the small sample size, further study is needed. Targeting mature DC with viral vectors may render cellular vaccines more functional and immunogenic.

## Supporting Information

Figure S1 **Characterization of T cell responses for volunteer RV138.08 (IM group).** Epitope mapping using a peptide matrix ELISPOT assay with in vitro expanded (pIVS) PBMC revealed that peptides 75 and 76 were positive. The main epitope recognized was confirmed to be MN Env peptide #76 (CTRPNYNKRKRIHIG Env 296–310, HXB2 location). In an ICS assay, 41.5% and 0.61% of the CD3/CD8 positive T cells in the pIVS preparation were positive for IFN-γ staining to p76 pulsed v.s. unpulsed autologous TBC (data not shown). The B cell line was HLA typed and the class I HLA type of this individual is: A*02011, A*74(01,02), B*0703, B*4001, Cw*0304s, Cw*0802s. As predicted, the Env #75/76 peptide overlap contains a motif matching the binding capacity of HLA-B*0703 with the minimal epitope predicted to be RPNYNKRKRI. Peptide 76 was used to continue in vitro stimulation of the culture and further characterized in 51Cr-release CTL and ICS assays. (A) CTL capacity of the line was confirmed by examination of cross-subtype reactivity to rVV expressed Envelope proteins representing subtypes A, B, C, D, CRF01_AE, F, G and H. As predicted from the sequences there was no cross-subtype reactivity and only the autologous MN Env was recognized. (B) The CTL assay also confirmed that both peptide #75 and #76 pulsed TBC were lysed by this line. The CTL line was also able to secrete granzyme B as detected by an ELISPOT assay (data not shown). (C) The peptide 76 reactive T cell line could recognize the original Env peptide pools that were use for direct ex vivo ELISPOT screening in an ICS format assay. The predicted Env pools 3 & 4, which contain the peptides 75–76 were recognized at a frequency of 41 and 51% respectively by ICS. For the Becton Dickenson Env peptide pool (containing 160 HIV-1 Env peptides) recognition was only 10.2%. The individual peptide #76 was recognized at a frequency of 56%. vSC8; control vaccinia, us; unstimulated, PP; peptide pool.
(TIF)

Protocol S1 **Trial Protocol.**
(PDF)

Checklist S1 **CONSORT Checklist.**
(DOC)
