# Supplementary figures and images for: A Double-Blind Randomized Phase I Clinical Trial Targeting ALVAC-HIV Vaccine to Human Dendritic Cells
Source: PLoS One. 2011 Sep 16;6(9):e24254. doi: 10.1371/journal.pone.0024254 (PMC3174939; doi:10.1371/journal.pone.0024254)

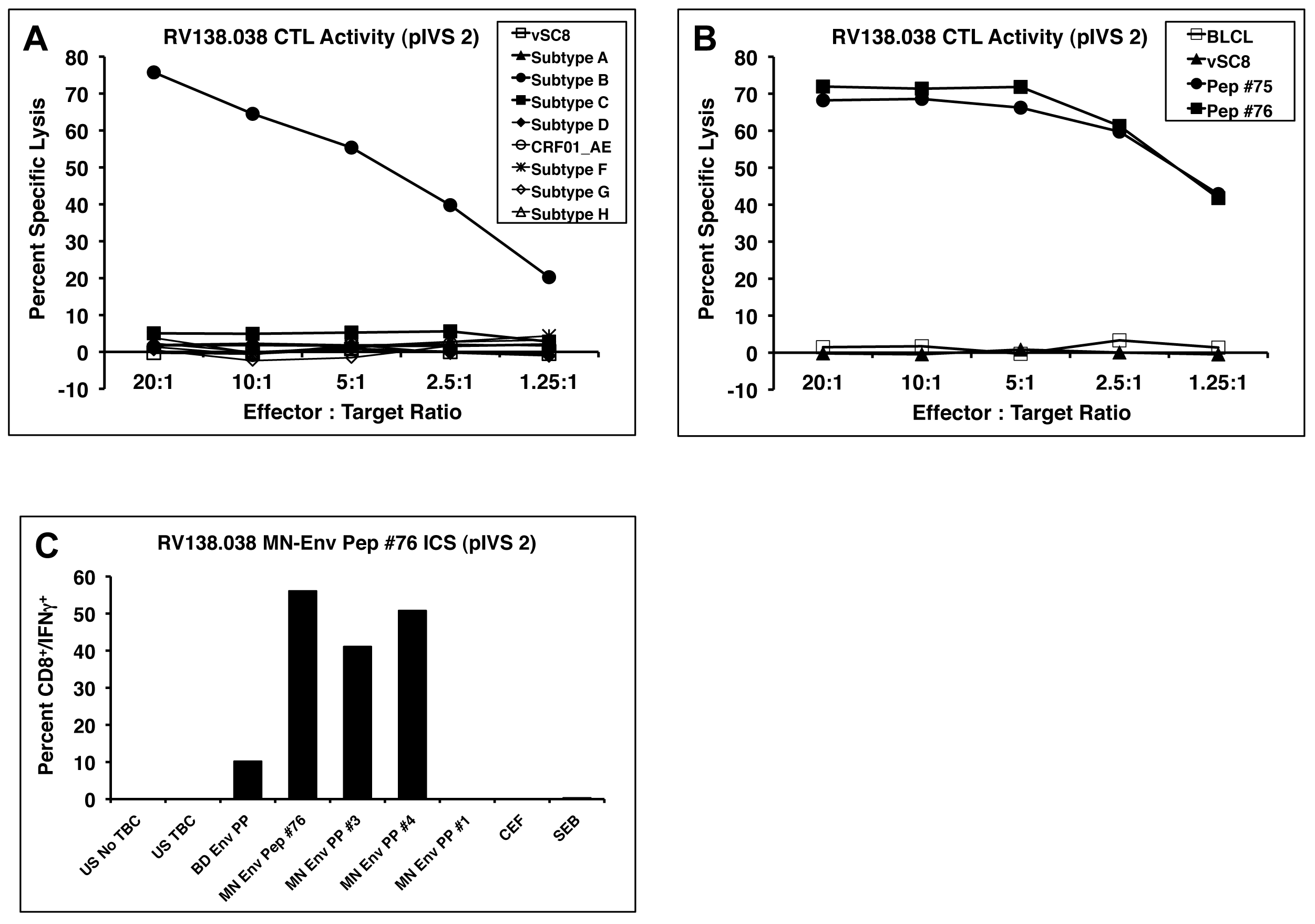

Supplement: Figure S1 — Characterization of T cell responses for volunteer RV138.08 (IM group). Epitope mapping using a peptide matrix ELISPOT assay with in vitro expanded (pIVS) PBMC revealed that peptides 75 and 76 were positive. The main epitope recognized was confirmed to be MN Env peptide #76 (CTRPNYNKRKRIHIG Env 296–310, HXB2 location). In an ICS assay, 41.5% and 0.61% of the CD3/CD8 positive T cells in the pIVS preparation were positive for IFN-γ staining to p76 pulsed v.s. unpulsed autologous TBC (data not shown). The B cell line was HLA typed and the class I HLA type of this individual is: A*02011, A*74(01,02), B*0703, B*4001, Cw*0304s, Cw*0802s. As predicted, the Env #75/76 peptide overlap contains a motif matching the binding capacity of HLA-B*0703 with the minimal epitope predicted to be RPNYNKRKRI. Peptide 76 was used to continue in vitro stimulation of the culture and further characterized in 51Cr-release CTL and ICS assays. (A) CTL capacity of the line was confirmed by examination of cross-subtype reactivity to rVV expressed Envelope proteins representing subtypes A, B, C, D, CRF01_AE, F, G and H. As predicted from the sequences there was no cross-subtype reactivity and only the autologous MN Env was recognized. (B) The CTL assay also confirmed that both peptide #75 and #76 pulsed TBC were lysed by this line. The CTL line was also able to secrete granzyme B as detected by an ELISPOT assay (data not shown). (C) The peptide 76 reactive T cell line could recognize the original Env peptide pools that were use for direct ex vivo ELISPOT screening in an ICS format assay. The predicted Env pools 3 & 4, which contain the peptides 75–76 were recognized at a frequency of 41 and 51% respectively by ICS. For the Becton Dickenson Env peptide pool (containing 160 HIV-1 Env peptides) recognition was only 10.2%. The individual peptide #76 was recognized at a frequency of 56%. vSC8; control vaccinia, us; unstimulated, PP; peptide pool. (TIF) [file pone.0024254.s001.tif]
