# Supplementary material for: A Double-Blind Randomized Phase I Clinical Trial Targeting ALVAC-HIV Vaccine to Human Dendritic Cells
Source: PLoS One. 2011 Sep 16;6(9):e24254. doi: 10.1371/journal.pone.0024254 (PMC3174939; doi:10.1371/journal.pone.0024254)
Supplement: Protocol S1 — Trial Protocol. (PDF) [file pone.0024254.s002.pdf]

CLINICAL STUDY PROTOCOL

RV 138: A Phase I study of Aventis Pasteur Live Recombinant ALVAC-HIV (vCP205, HIV-1 env/ gag/pol) in Seronegative Adults Administered (1) Subcutaneously via *ex vivo* Transfected, Autologous Dendritic Cells, (2) Intradermally, or (3) Intramuscularly.

Principal Investigator:
Mary A. Marovich, MD, DTMH
Walter Reed Army Institute of Research
13 Taft Ct., Ste 200
Rockville, MD, 20850
Tel: 301-251-8337
Fax: 301-762-4177
mmarovoich@hivresearch.org

Sponsor (Study IND Holder):

Office of the Surgeon-General
Department of the Army

Sponsor's Representative:
COL Jerome F. Pierson
Commander, U.S. Army Medical Material
Development Activity (USAMMDA)
HQ, USAMRMC
ATTN: MCMR-UMR
622 Neiman Street
Fort Detrick, MD 21702-5012
USA

Manufacturer:

Aventis Pasteur
1 Discovery Drive
Swiftwater, PA. 18370
USA

Project Assurance Number: 20057
IND number: 9414

Clinical Site: U.S. Military HIV Vaccine Program Clinical Study Site, 1600 East Gude Drive, Rockville, MD 20850

Clinical Study Coordinator: Kathleen Duffy, RN

Medical Monitor: Nelson L. Michael, MD

Confidential

The information contained herein is the property of Office of the Surgeon-General Department of the Army and Aventis Pasteur, and may not be reproduced, published, or disclosed to others without written authorization.

PROTOCOL SUMMARY

**Title of study:** A Phase I study of Aventis Pasteur Live Recombinant ALVAC-HIV (vCP205 HIV-1, env/gag/pro) in HIV Seronegative Adults Administered (1) Subcutaneously via *ex vivo* Transfected, Autologous Dendritic Cells, (2) Intradermally, or (3) Intramuscularly
**Sponsor (IND Holder):** Office of the Surgeon-General Department of the Army (OTSG)
**Project Phase**: Phase I prospective randomized, double-blinded, placebo-controlled trial with 3 arms describing the safety and immunogenicity of alternative routes of delivery of vCP205:<br><br>Group I. subcutaneous reinjection of *ex vivo* generated autologous dendritic cells transfected with ALVAC-HIV (vCP205) and pulsed with KLH (control antigen)<br>Group II. intradermal injection of ALVAC-HIV (vCP205)<br>Group III. intramuscular injection of ALVAC-HIV (vCP205) as the standard method of delivery
**Study Design**: A Phase I study<br>All volunteers (36 low-risk HIV seronegatives) will undergo leukopheresis prior to vaccination and 2 weeks after the final vaccination. Their cells will be cryopreserved in aliquots and will serve as a source of (prevaccination or postvaccination) cells throughout the study and will reduce the number and volume of blood draws.<br>The study is divided into 3 Groups of 12 volunteers each. Each group will have the same vaccination schedule of 0,1,3 and 6 months.<br>Group I. will have autologous DC generated from prevaccination leukopheresis cells. Laboratory manipulation of the cells will be done using GLP.<br>There will be 8 volunteers who will receive ALVAC-HIV infected DC pulsed with KLH and 4 volunteers who will receive KLH pulsed DC only. The KLH will be added for the first vaccination only. For each subject, the maximal number of available DC will be reinjected within an anticipated range of 1.5×10^6-6×10^6 cells in 0.4 ml volume. Please see **Appendix A** for details on DC preparation, DC transfection, KLH pulsing, release criteria and determination of percent transfection.<br>Group II. ALVAC-HIV (vCP205) intradermally (ID), ~$10^{6.5}$ $TCID_{50}$ reconstituted in 1 ml of sterile water and delivered in 4 x 0.125 ml intradermal injections. There will be 8 vaccine recipients and 4 placebo (saline) recipients.<br>Group III. ALVAC-HIV (vCP205) intramuscularly (IM), ~$10^{6.5}$ $TCID_{50}$ reconstituted in 1 ml of sterile water and delivered as a single 1.0 ml intramuscular injection. There will be 8 vaccine recipients and 4 placebo (saline) recipients.<br>Please note: the ~$10^{6.5}$ $TCID_{50}$ dose is given as an approximate dose because we will be using a new "lot" of vCP205. This is a representative amount of vaccine in a single use vial used in previous trials.
**Subjects**: 36 healthy, HIV seronegative adults, 18 to 55 years of age (< 10% will be > 50 yrs.old), available for at least 15 months of follow-up from the time of screening.<br>Group I. 12 volunteers, 8 receiving active agent, ALVAC-HIV infected DC pulsed with KLH, and 4 receiving KLH pulsed DC only (control DC).<br>Group II. 12 volunteers, 8 receiving active agent (ALVAC-HIV), 4 receiving placebo.<br>Group III. 12 volunteers, 8 receiving active agent (ALVAC-HIV), 4 receiving placebo.

Study Site:
Clinical study site: U.S. Military HIV Vaccine Program Clinical Study Site, 1600 East Gude Drive, Rockville, MD 20850

Lab study site: U.S. Military HIV Vaccine Program Clinical Study Site, 1600 East Gude Drive, Rockville, MD 20850

GLP facility: U.S. Military HIV Vaccine Program Clinical Study Site, 13 Taft Ct, Suite 200 Rockville, MD 20850

CLINICAL INVESTIGATORS:

Merlin Robb, MD
US Military HIV Research Program
1600 E. Gude Drive
(301) 251-8302
(301) 762-7460

Sybil A. Tasker, MD
National Naval Medical Center
8901 Wisconsin Ave.
Bethesda, MD 20889
(301) 295-5246
(301) 295-2831

Shirley Lecher, MD
US Military HIV Research Program
13 Taft Court
Rockville, MD 20850
301-251-7769

Darrell Singer, MD, MPH
US Military HIV Research Program
13 Taft Court
Rockville, MD 20850
301-251-8315

LABORATORY INVESTIGATORS:

Deborah L. Birx, M.D.
Global AIDS Program (GAP)
HHS/CDC/NCHSTP
Corporate Square Building 8
Atlanta, GA 30329
404-639-8320

Thomas VanCott, PhD
Director, Product Develoment
Advanced BioScience Laboratories
5510 Nicholson Lane
Kensington, MD 20895
301-816-5248

Josephine Cox, PhD
Henry M. Jackson Foundation
13 Taft Court
Rockville, MD 20850
301-251-8360

Maryanne Vahey, PhD, DAC
Deputy Director
Division of Retrovirology
Walter Reed Army Institute of Research
1600 East Gude Drive
Rockville, MD 20850
(301) 251-5058

Victoria R. Polonis, PhD, DAC
Division of Retrovirology
Walter Reed Army Institute of Research
13 Taft Court
Rockville, MD 20850
(301) 251-8308

Ralph M. Steinman, MD
Laboratory of Cellular Physiology and Immunology
The Rockefeller University
1230 York Ave.
New York NY 10021
(212)-327-7763

Nina Bhardwaj, MD, PhD
Laboratory of Cellular Physiology and Immunology
The Rockefeller University
1230 York Ave.
New York NY 10021
(212)-327-7763

Madhav Dhodapkar, MD
Laboratory of Cellular Physiology and Immunology
The Rockefeller University
1230 York Ave.
New York NY 10021
(212)-327-7763

Route of Administration: *Ex vivo* generated and transfected, autologous dendritic cells will be injected into the subcutaneous tissue of the upper arm with a tuberculin syringe. Vaccine via intradermal injection will be injected into the dermis of the volar skin of the forearm. Intramuscular injections will be administered into the deltoid muscle of the arm.

IMMUNIZATION SCHEDULE

Subcutaneous Administration of vCP205 via transfected, autologous dendritic cells

			Months			
Group	Vaccine/ Placebo*	Subcutaneous Route	0	1	3	6
I	8/4	DC	DC-vCP205 KLH pulsed or KLH pulsed DC only	DC-vCP205 or DC only	DC-vCP205 or DC only	DC-vCP205 or DC only

Vaccine = vCP205 transfected DC pulsed with KLH, *Placebo = DC pulsed with KLH only (control DC) delivered in 2 x 0.2 ml subcutaneous injections. The KLH is used in the first vaccination only.
vCP205 = ~$10^{6.5}$ $TCID_{50}$ (1 vial) reconstituted in1 ml sterile water/ 2-3 x 10^6 DC

Intramuscular or Intradermal Administration of vCP205 via injection

			Months			
Group	Vaccine/ Placebo*	Route	0	1	3	6
II	8/4	Intradermal	vCP205	vCP205	vCP205	vCP205
III	8/4	Intramuscular	vCP205	vCP205	vCP205	vCP205

ID= ~$10^{6.5}$ $TCID_{50}$ (1 vial) reconstituted in1 ml sterile water, delivered in 4 x 0.125 ml intradermal injections (0.5 ml total dose).
IM= ~$10^{6.5}$ $TCID_{50}$ (1 vial) reconstituted in1 ml sterile water, delivered in a single 1 ml intramuscular injection (1.0 ml total dose).
[1]Placebo = sterile saline

STUDY ENDPOINTS

Safety and Tolerability: Tabulation of adverse effects and laboratory abnormalities: All subjects will be observed for 1 hour following immunizations for evidence of immediate local and systemic reactions. They will be instructed to watch for local and systemic reactions for 7 days post-immunization and will be evaluated by an investigator if significant symptoms are reported. Routine measurements of hematology, serum chemistry, and urinalysis laboratory tests will be performed 14 days after each vaccination.

Immunogenicity: Description of the following primary measures of immunogenicity.

• Lymphocyte proliferation responses to specific vaccine component antigens
(which includes gp160 MN/LAI protein from Aventis Pasteur and p24 LAI protein from MicroGeneSys) and to the control antigen KLH (for DC arm only).
• Cytotoxic T-lymphocyte (CTL) responses against vaccine component antigens expressed in a recombinant poxvirus vector system (expressing HIV env MN antigen, gag and pol LAI antigens) in standard bulk chromium release assays.
• ELISPOT analysis for gamma-interferon production from bulk PBMCs in response to vaccine antigen stimulation (or other relevant antigens as they become available) in an overnight culture system.
• Enumeration and characterization of antigen specific gamma-interferon producing cells using flow cytometry.

BLOOD REQUIREMENT: 639.6 ml for all vaccine recipients over a 15-month period.

INTERIM AND FINAL ANALYSES: Preliminary safety analysis will be performed after the first and second immunizations are completed. A final analysis will occur after all visits are complete.

TABLE OF CONTENTS

1. LIST OF ABBREVIATIONS AND DEFINITIONS OF TERMS 11

2. ETHICS 12

2.1 Institutional Review Board (IRB) 12

2.2 Informed Consent 12

3. INVESTIGATORS AND STUDY ADMINISTRATIVE STRUCTURE 12

4. BACKGROUND AND RATIONALE 13

5. STUDY OBJECTIVES 16

6. INVESTIGATIONAL PLAN 17

6.1 Overall Study Design and Plan 17

6.2 Rationale for the Study Arms: 18

6.3 Assessment of Immunogenicity 18
6.3.1 Primary Measures of Immunogenicity--All Subjects 18
6.3.2 Ancillary Immunogenicity Studies 19

6.4 Selection of Study Populations 19
6.4.1 Inclusion Criteria: 19
6.4.2 Exclusion Criteria: 20
6.4.3 Removal of Subjects from Trial or Assessment 21

6.5 Administration of Vaccines 21
6.5.2 Identity of Investigational Products 23
6.5.2.1 Vaccine Supplies 23
6.5.2.2 Procedure for Reconstitution 23
6.5.2.3 Method of Dosage Administration 24
6.5.2.4 Disposition 24
6.5.2.5 Precautions to be Observed in Administering Study Vaccine 25
6.5.3 Method of Assigning Subjects to Study Groups 25
6.5.4 Controls/Blinding 25
6.5.5 Prior and Concomitant Medications/Vaccines 25
6.5.6 Study Procedures 26

6.6 Study Variables and Their Measurement 30
6.6.1 Immunogenicity Variables 30
6.6.1.1 Specimen Handling and Processing 31
6.6.1.2 Sample collection 31
6.6.2 Safety Variables 32
6.6.2.1 Post-immunization Events Monitored as Possible Reactions to Vaccine 32
6.6.2.2 Stopping Criteria 33

6.6.2.3 Other Adverse Events 33
6.6.2.4 Diagnostic Algorithm to Differentiate Immunogenicity from HIV Infection: 35
6.6.2.5 Management of Volunteers with HIV-1 Infection During the Trial 36

6.7 Procedural Flow Chart 37

6.8 Volunteer's Vaccine Schedule 38

6.9 Statistical Methods and Determination of Sample Size 39
6.9.1 Statistical Plan: Design and Sample Size Calculation 39
6.9.2 Safety 39
6.9.2.1 Postimmunization Reactions (Local and Systemic) 39
6.9.2.2 Other Adverse Events 40
6.9.2.3 Clinical Laboratory 40
6.9.3 Immunogenicity 40

7. COMPENSATION, INSURANCE, INDEMNITY 41

7.1 Information on compensation, insurance and indemnity 41

7.2 Advocacy 41

8. VACCINE ACCOUNTABILITY 41

9. LABORATORY REQUIREMENTS 42

10. CASE REPORT FORMS 42

11. STUDY MONITORING AND COMPLIANCE 42

12. PROTOCOL DEVIATION REPORTING 42

13. RETENTION OF RECORDS 43

14. USE OF INFORMATION AND PUBLICATION 43

15. PROTOCOL AMENDMENTS 44

16. REFERENCES 45

17. SIGNATURE PAGE 49

18. APPENDICES 50

18.1 STATEMENT OF OBLIGATIONS OF SPONSOR/MONITOR AND CLINICAL INVESTIGATOR 50

18.2 TRIAL CONSENT FORM 53

18.3 TEST OF UNDERSTANDING AND COMMITMENT 54

18.4 PREGNANCY REPORTING 55

18.5 MEDICAL MONITOR 56

18.6 TOXICITY GRADING SCALE 57

1. LIST OF ABBREVIATIONS AND DEFINITIONS OF TERMS

Abbreviation/Acronym	Definition
ACD	acid citrate dextrose
AIDS	acquired immunodeficiency syndrome
ALT	alanine transferase
ANA	antinuclear antibody
ANOVA	analysis of variance
Ab	antibody
BHK	hamster kidney cell line
CBC	complete blood count
CBER	Center for Biologics Evaluation and Research (FDA)
COSTART	coding symbols for a thesaurus of adverse reaction terms
CPM	counts per minute
CRF	case report form
CTL	cytotoxic T lymphocyte
DC	dendritic cell
DCAC	Data Coordinating and Analysis Center
DCDN	dendritic cell donor number
DNA	deoxyribonucleic acid
DSMB	Data and Safety Monitoring Board
EDTA	ethylenediaminetetraacetic acid
ELISA	enzyme linked immunosorbent assay
FDA	US Food and Drug Administration
FTA	fluorescent treponemal antibody
GLP	Good Laboratory Practice
GMT	geometric mean titer
GM-CSF	granulocyte monocyte-colony stimulating factor
GTMR	greater than minimal risk
HBsAg	hepatitis B surface antigen
HIV and HIV-1	human immunodeficiency virus, type 1
HJF/HMJF	Henry M. Jackson Foundation for the Advancement of Military Medicine
HLA	human leukocyte antigen
ICC	intracellular cytokine cytometry
ID	intradermal
IL-4	interleukin-4
IM	intramuscular
IRB	Institutional Review Board
KLH	keyhole limpet hemocyanin
LPA	lymphocyte proliferation assay
LSI	Lymphocyte stimulation index
MCM	monocyte conditioned media
MHC	major histocompatibility complex
MOI	Multiplicity of Infection
MTF	Medical Treatment Facility

NAb	neutralizing antibody
NIH	National Institutes of Health
OTSG	Office of the Surgeon General, Department of the Army
ORM	Office of Research Management
PBMC	peripheral blood mononuclear cells
PCR	polymerase chain reaction
PIN	personal identification number
RBC	red blood cell count
RF	rheumatoid factor
RIBA	recombinant immunoblot assay
RNA	ribonucleic acid
RPR	rapid plasma reagin
SIV	simian immunodeficiency virus
TT	tetanus toxoid
TBD	to be determined
$TCID_{50}$	cell culture infectious dose (50%)
TCLA	tissue culture laboratory adapted
UA	urinalysis
USAMMDA	U.S. Army Medical Materiel Development Activity
USAMRMC	U.S. Army Medical Research and Materiel Command
USMHRP	U.S. Military HIV Research Program
WB	Western blot
WBC	white blood cell count
WFI	water for injection
WRAIR	Walter Reed Army Institute of Research

2. ETHICS

2.1 Institutional Review Board (IRB)

The principal investigator agrees to provide the IRB with all appropriate material, including the informed consent documents. This trial will not be initiated until appropriate IRB approval of the protocol, informed consent document, and all recruiting materials have been obtained in writing by the investigator and the sponsor has received copies. Appropriate reports on the progress of the study by the principal investigator will be made to the IRB and the sponsor in accordance with applicable government regulations and in agreement with policy established by the sponsor.

2.2 Informed Consent

A properly executed written informed consent, in compliance with the Declaration of Helsinki, guidelines of the Council of International Organization of Medical Sciences (CIOMS) and US law 21 CFR 50, shall be obtained from each subject prior to entering the subject into trial or prior to performing any unusual or non routine procedure that involves risk to the subject. The investigator shall provide a copy of the IRB-approved informed consent to the subject and a signed copy shall be maintained in the subject's record file. Attention is directed to the basic elements that are required to be incorporated into the informed consent under US Federal Regulations for Protection of Human Subjects (21 CFR 50.25[a]). Additional elements of informed consent if appropriate, must be included in the informed consent document (21 CFR 50.25[b]).

3. INVESTIGATORS AND STUDY ADMINISTRATIVE STRUCTURE

Principal Investigator: Mary A. Marovich, MD, DTMH
13 Taft Court, Suite 200
Rockville, MD 20850
Ph (301) 251-8337
Fax (301) 762-4177

Medical Monitor: Nelson L. Michael, MD

4. BACKGROUND AND RATIONALE

Introduction

The World Health Organization estimates that HIV-1 currently infects 33.6 million people (including 1.2 million children) worldwide and over 5 million have developed AIDS [1]. New HIV-1 infections occur at a rate of approximately 16,000 per day[2], 95% in underdeveloped countries[1], where anti-retroviral therapies will be severely limited.[2] An obvious solution to this worldwide epidemic is an effective preventive vaccine for HIV. Despite intense investigation such a vaccine has yet to be developed.

Cytotoxic T cell (CTL) responses are important in viral destruction and thereby in the protective immune response to viral infection. The protection offered by this cellular immune response is mediated both by direct lysis of HIV-targets and also by soluble factors (anti-viral chemokines and cytokines) produced by CTL. Current HIV vaccines do not consistently elicit strong cytotoxic T cell (CTL) responses.[54-61] This limited immune response could be attributed to intrinsic properties of the antigens, suboptimal antigen presentation, or simply that the vaccine is not reaching the appropriate cells to initiate a robust immune response. This exploratory, proof-of-concept study seeks to insure adequate delivery of the vaccine to important antigen presenting cells.

The body has a system of specialized antigen presenting cells, termed dendritic cells [DCs], that are particularly effective in stimulating primary T-cell dependent immunity. These cells are located in key sites within the body where they act as sentinels, frequently encountering foreign antigens (skin, mucosa, gastrointestinal tract). Once they capture an antigen, they undergo cellular changes that allow them to migrate to the local draining lymph node where they can effectively present this antigen to T cells. The efficacy of this cell type is clear from four lines of evidence: i] relatively small numbers of DCs are sufficient to stimulate strong T cell responses [3-7] ii] DCs activate quiescent T cells including naive or unprimed lymphocytes [7-10] ; iii] DCs induce CD4+ helper and CD8+ killer T cells *in vivo* in animals [11-22] ; iv] DCs have specialized receptors and intracellular vacuoles for enhancing antigen uptake and presentation [23-27]. For these reasons, DCs are now considered to be "nature's adjuvant" for the development of immunity to antigens [28-30]. It has long been considered desirable to use antigen-bearing DCs as adjuvants for active immunotherapy in humans, particularly to increase host resistance to tumors and certain viral infections. This study seeks to insure adequate delivery of the vaccine to these important antigen-presenting cells (DC) and to assess the effects of this direct targeting technique. We propose to use antigen loaded-DC as a primary preventative measure as it is generally preferable to prevent disease than to attempt to cure it once it has taken hold or disseminated within the human body (e.g. cancer, AIDS).

We can obtain potent DCs in large numbers from peripheral blood samples, by applying cytokines to precursors within the monocyte fraction. We will draw blood from prospective donors and isolate their mononuclear cells using a standard ficoll centrifugation technique. These mononuclear cells are then placed into a plastic dish and incubated for 2 hours and allowed to adhere to the surface.

The non-adherent cells are washed away and the remaining cells (enriched monocyte fraction) are treated with clinical grade recombinant human Granulocyte Monocyte-Colony Stimulating Factor (rHuGM-CSF) and recombinant human Interleukin-4 (rHuIL-4) for 5 days. These cells are then harvested from the wells and re-plated into another plastic dish and treated with autologous monocyte conditioned medium (MCM). This MCM will be generated from the donor early in the study (from initial leukopheresis) and is used to mature the DC as a means of increasing the immunostimulatory capacity of the cells and in stabilizing their phenotype.

One of the main goals of this study is to transfect the DCs with a canarypox expressing HIV immunogens and test their capacity to induce an immune response following subcutaneous administration. Specifically, we hope to see a robust increase in the cellular immune response to the vaccine delivered via dendritic cells when compared to the standard intramuscular route. Intradermal delivery may be a more straightforward way to efficiently target the DC *in vivo* and thereby avoid *ex vivo* manipulation. Several existing vaccines are specifically designed to be given intradermally (rabies, cholera, and hepatitis B) for maximal humoral immunogenicity [31-33]. Other pox vaccines delivered intradermally have induced broader immune responses when compared to intramuscular delivery [34]. Skin dwelling DCs reside mainly in the epidermis and dermis and the ID injection of the vaccine may enhance its delivery to the local DC system. If the ID route facilitates vaccine access to the skin DCs, we would expect an improvement in immune response over the usual IM route. Therefore, we expect to see the typical modest cellular response rate (~30-50%) to the standard IM route [54-61], an improved response to the intradermal delivery route, and the maximal immune response to the directly targeted (*ex vivo*) pulsed DC.

Recently, DC have been enriched from human blood and used to immunize lymphoma patients to the Ig receptor that is expressed by their (autologous) tumor, as well as to an irrelevant protein, keyhole limpet hemocyanin or KLH [35], as a means of cancer therapy. KLH is an immunogenic protein that induces the production of anti-KLH antibodies and an anti-KLH cellular immune response. Its use allows a method to monitor the induction of primary immunity because it is a neoantigen, i.e. one that the human immune system has never encountered. It is frequently used in conjunction with DC immunotherapy [35-37,61,64,65] as an independent means of tracking the primary immune response and as possibly providing “help” for the development of anti-tumor immunity**.** These DC were administered I.V. and no toxicities were observed. Beneficial clinical responses were reported in all DC-immunized patients (3/4 tumor shrinkage, 1/4 tumor stability-no progression). Since this initial successful study in humans with B-cell lymphoma, other tumor antigens have been used to pulse *ex vivo* generated DC for re-infusion with beneficial results [36-38]. Patients with malignant melanoma have been reported to benefit from tumor-Ag pulsed DC immunization [36]. Multiple pulsed DC injections were given with little to no toxicity (limited to mild, local reactions at the injection site). Subsequent immunologic monitoring after DC immunization showed antigen specific delayed type hypersensitivity (DTH) responses in 11 of 16, and in some of these responders, peptide specific CTL could be recovered from skin biopsies. Importantly, 5 of 16 patients had tumor regression including 2 complete responses lasting greater than 15 months. Another more recent melanoma trial which uses mature, autologous DC pulsed with a melanoma peptide, Mage-3A1, showed expansion of tumor specific CD8+ CTL precursors and clinical responses to that immunological phenomenon [62]. The latter manifested as metastasis regression in various sites including the skin where local biopsies showed heavy infiltrate of tumor specific CD8+ cells. Whereas non-regressing lesions lacked CD8+ T cells as well as Mage-3A1 mRNA expression which explains the “escape” of antigen negative metastases. Other reports of DC based anti-tumor immunotherapy indicate clinical responses in advanced, widely metastatic renal cell carcinoma and refractory prostate carcinoma without significant adverse toxicities and positive responses in 25-30% of those immunized [37-38]. Many ongoing clinical trials using a broad array of tumor derived epitopes include: tumor lysates, defined tumor antigens, tumor peptides, tumor cell-DC conjugates, tumor cell RNA, and apoptotic tumor cells will address optimal loading methods.

Since the normal trafficking pattern of DCs *in vivo* is to move from the subcutaneous tissues via the lymphatics to the T cell areas of draining lymph nodes [44-49], we propose to optimize immune

responses by reinfusing autologous DCs subcutaneously. With the increasing evidence for anti-tumor activity of tumor-antigen pulsed DCs, there is now a major interest to direct DC based immunization toward the development of prophylactic vaccines. Previous work using influenza infected DC has shown specific induction of human CD8+ CTL responses [45-47]. Recently, researchers from the Rockefeller University have published very promising results showing that *ex vivo* generation of autologous mature DC pulsed with an influenza derived peptide, tetanus toxoid and KLH, and reinfused subcutaneously are safe and much more immunogenic than the various peptides or antigens injected alone (without DC). These specialized cells are fully capable of generating rapid cellular immune responses, after a single injection, within both the CD4+ and CD8+ T cell compartments [50].

Preliminary data is accumulating from ongoing collaborative efforts between WRAIR scientists and the same aforementioned researchers at Rockefeller using primates and canarypox-SIV infected DC (SIV is the simian immunodeficiency virus often studied in nonhuman primates to model HIV infection). Preliminary safety data is very reassuring in that 12 monkeys (Rhesus macaques) have been injected with autologous DC transfected with the canarypox-SIV vaccine without serious adverse events and excellent tolerability. The immunogenicity data suggest that canarypox-SIV infected monkey DC are capable of rapidly inducing proliferative T cell responses. Additionally, unpublished but submitted data from the same Rockefeller group now clearly demonstrate that canarypox infection of mature human DCs induces both CD4+ and CD8+ T cell responses *in vitro* in HIV seropositives. [66] These results suggest that when targeted to DCs, canarypox has the potential to stimulate both CD4+ and CD8+ components of the anti-HIV immune response. [66] No such *in vivo* studies have been performed in human populations with canarypox-HIV-1 immunogens and DC. This study would be one of the first to test an existing and safe HIV vaccine candidate (vCP205) directly targeted onto autologous DC for reinfusion. This is a biological proof-of-concept study as this vaccine strategy is not considered widely deployable. If direct targeting of autologous DC with an HIV immunogen shows positive results, then delivery methods of HIV antigens targeted to dendritic cells *in vivo* would be greatly considered.

One of the reassuring features of this emerging DC vaccine technology is the remarkable safety profile. There has been no significant acute toxicity related to DC injections in over 100 patients treated [63]. Most acute reactions are minor local irritations at the site of injection after multiple vaccinations and may be attributable to recall phenomenon (i.e. tetanus) as was reported in a recent melanoma trial [62]. The potential concern for autoimmunity is acknowledged by all investigators. We plan to evaluate the possibility of such a development by checking baseline autoimmune antibodies (ANA, Rheumatoid Factor, and anti-thyroid antibodies) and will continue to monitor these during the conduct of the trial. It is possible that the lack of development of autoimmunity using DC-based immunization strategies may be the fact that DC are likely involved in the deletion of autoreactive T cells *in vivo* [63].

Canarypox HIV (ALVAC-HIV) Vaccine Candidate

The Aventis Pasteur ALVAC-HIV "candidate" vaccine (vCP205) is a preparation of recombinant canarypox virus expressing the products of the HIV-1 *env*, *gag*, and *pro* genes. The genes are inserted into the canarypox C3 locus under the control of the vaccinia virus H6 and I3L promoters. The sequences are derived from the subtype B HIV-LAI strain, but gp120 from HIV-MN (also subtype B) replaces the LAI gp120. Co-expression of gag and env, and appropriate gag processing by protease, in a vaccinia expression system, results in the formation of virus-like-particles that bud from the cell membrane.[50] Thus, ALVAC-infected cells likely present Env and Gag proteins in a near-native conformation. In addition, intracellular processing of foreign HIV-1 proteins via the MHC class I pathway facilitates stimulation of cytotoxic T-lymphocytes. The vCP205 construct also contains a deletion in a highly immunogenic portion of the gp41 envelope transmembrane region. Since the vast majority of HIV-1-infected subjects make antibody to this region, the lack of antibody in vCP205 recipients will make it possible to distinguish between vaccine recipients and HIV-1-infected subjects.

The percent of subjects with cross-reactive CTL and the optimal immunization regimen remain to be determined.[67] These data raise the possibility that subtype B based vaccine products that elicit CTL may be effective in preventing HIV-1 infection or disease caused by non-subtype B viruses. Extensive testing of ALVAC candidate vaccines encoding a variety of different viral products have provided no evidence of toxicity in mammals.[50] In AVEG012 there was an average increase in canarypox antibody, from baseline, of 70- to 120-fold. These titers were stable after the second of four vaccinations with vCP125.[51]

Previous studies with ALVAC-HIV constructs.

Eighteen phase I trials of Aventis Pasteur ALVAC-HIV candidate vaccines alone or boosted with soluble protein have been conducted, or are ongoing, in France and in the United States.[54-61] The vaccine has been well tolerated. No serious adverse events attributable to the ALVAC vaccine have been reported in over 1500 HIV seronegative subjects receiving an ALVAC construct (Aventis Pasteur, unpublished data). The experience with vCP205 is accumulating and data indicate that the vaccine induces HIV-specific CTL responses in approximately 30-50% of volunteers.[54-61] However, these CTL responses were sporadic and not sustained in most responders. When given alone, ALVAC-HIV induces limited neutralizing antibody responses in many or most volunteers after 3 of 4 immunizations; the neutralizing antibody titers are modest. While neutralizing antibodies do not appear to be active across HIV-1 subtypes, CTL activity can be demonstrated across HIV-1 subtypes in some subjects.[67]

AVENTIS PASTEUR ALVAC-HIV (vCP205) is grown in chick embryo fibroblasts derived from pathogen-free chicken eggs. The harvested cells are pelleted by centrifugation, suspended in culture medium, and disrupted by sonication. Cell debris is largely removed by centrifugation. An equal volume of lactoglutamate is added to the supernatant; this represents the Master Seed Lot which is stored at ≤ –35˚C. The Working Seed Lot is derived from the Master Seed Lot according to the same production scheme and stored at ≤ –35˚C. The Production Lot is prepared from the Working Seed Lot. After culture on chick embryo fibroblasts, the cells are harvested by centrifugation, and the pellet is suspended in Tris buffer. Cells are disrupted by sonication and the debris is removed by centrifugation. An equal volume of stabilizer is added to the supernatant, and the suspension is filtered through a 5 µm membrane and stored at ≤ –35˚C. The Production Lot is thawed, and an equal volume of freeze-drying medium is added to produce the Final Bulk Vaccine. The product is immediately transferred to the Filling Department, and the Filled Vaccine is prepared and lyophilized. Final containers are stored at –20˚C at Marcy l'Etoile, France and will be stored between +2˚ to +8˚C at the clinical site.

5. STUDY OBJECTIVES

The objectives of this study are to describe the safety and immunogenicity of ALVAC-HIV (vCP205) administered via 2 alternative delivery routes in an effort to improve the immunogenicity of this vaccine candidate as defined by improved cellular immune responses. It is a Phase I description of the acute safety and immunogenicity of ALVAC-HIV (vCP205) transfected autologous DC, or ALVAC-HIV (vCP205) delivered by the intradermal route. The internal referent will be the standard method of delivery via the intramuscular route. This will be the first study of vCP205 delivered ID or via autologous dendritic cells in HIV-seronegative humans.

6. INVESTIGATIONAL PLAN

6.1 Overall Study Design and Plan

This will be a phase I study in 36 low-risk, HIV-1 seronegative U. S. volunteers. The study is divided into three groups, all to describe safety and immunogenicity. In Group I, 12 volunteers will be given ALVAC-HIV (vCP205) transfected autologous dendritic cells pulsed with KLH (4 of these will receive KLH pulsed DC only) as a means of directly targeting these cells to enhance the immunogenicity of the vaccine. All four immunizations with the DC-vCP205 vaccines will be generated from the same initial leukopheresis blood cell product. DC precursors can be cryopreserved and prepared before each vaccination. In Groups II and III, 12 volunteers in each group, will be immunized with the exact same type of vaccine in current use, ALVAC-HIV (vCP205), but given by different routes (intradermal or intramuscular). Four volunteers will be given placebo (sterile saline) by the ID route and 4 volunteers by the IM route. In essence, we plan to describe the immune responses with directly injected ALVAC-HIV into the skin or muscle and ALVAC-HIV transfected DC injected into the subcutaneous space. All volunteers (all groups) will undergo leukopheresis before vaccination and 2 weeks after the final vaccination. Cells derived from the leukopheresis are crucial for establishing the dendritic cells for arm I of the study but will also be used to characterize and compare the immune responses against HIV within and between all three study arms. The advantage of the apheresis is that the large number of cells will permit comparison of novel CTL assays systems to accepted gold standard procedures and allow for the characterization of the sensitivity, specificity and reproducibility within and between arms. This pheresis process safely provides many more white blood cells than can be obtained from a routine venipuncture with minimal red blood cell loss. A new test for West Nile diagnosis was recently instituted in the WRAMC blood bank. All blood donors are required to have their blood tested for this infection. This may affect some volunteers in this trial, although no additional blood is drawn. It may be added to the list of routine blood tests already performed as part of the blood bank screening process.

This study will provide new, preliminary data regarding the respective immunogenicities of intradermal and *ex vivo* DC immunization with vCP205 relative to the moderately immunogenic IM route in normal human volunteers. The study is not designed to resolve the null hypothesis.

Subcutaneous Administration of vCP205 via transfected, autologous dendritic cells

			Months			
Group	Vaccine/ Placebo*	Subcutaneous Route	0	1	3	6
I	8/4	DC	DC-vCP205 KLH pulsed or KLH pulsed DC only	DC-vCP205 or DC only	DC-vCP205 or DC only	DC-vCP205 or DC only

Vaccine = vCP205 transfected DC pulsed with KLH, *Placebo = DC pulsed with KLH only (control DC) delivered in 2 x 0.2 ml subcutaneous injections. The KLH is used in the first vaccination only. vCP205 = ~$10^{6.5}$ $TCID_{50}$ (1 vial) reconstituted in1 ml sterile water/ 2-3 x 10^6 DC

Intradermal or Intramuscular Administration of vCP205 via injection

			Months			
Group	Vaccine/ Placebo[1]	Route	0	1	3	6

II	8/4	Intradermal	vCP205	vCP205	vCP205	vCP205
III	8/4	Intramuscular	vCP205	vCP205	vCP205	vCP205

ID= ~10 $^{6.5}$ $TCID_{50}$ (1 vial) reconstituted in1 ml sterile water, delivered in 4 x 0.125 ml intradermal injections (0.5 ml total dose).
IM= ~10 $^{6.5}$ $TCID_{50}$ (1 vial) reconstituted in1 ml sterile water, delivered in a single 1 ml intramuscular injection (1.0 ml total dose).
[1]Placebo = sterile saline

6.2 Rationale for the Study Arms:

Group I. Delivery of ALVAC-HIV vCP205 to autologous *ex vivo* generated DC. We will directly target the dendritic cell as a means of maximizing the antigen presentation of encoded HIV-1 genes within the vaccine vector. Theoretically, this arm would serve as the optimal antigen delivery method in this descriptive study. It will provide important conceptual information on the benefits of directly loading critical antigen presenting cells with vectors for HIV vaccine development. If the results indicate beneficial effects of directly targeted-DC, this type of study will lay the groundwork for future vaccine development with an emphasis on *in vivo* DC targeting. We will include the use of KLH as a control antigen in this arm (for the first injection only) as means of quality control to ensure that the canarypox transfection process is not inhibitory for DC function in some way. We can then reasonably use the anti-KLH (primary) immunity that would develop in the control DCs as an internal referent for this arm.

Group II. We propose to optimize the immunogenicity of this vaccine and augment the cellular immune response against HIV immunogens by using different methods of delivery. Other pox vaccines delivered intradermally have induced broader immune responses when compared to intramuscular delivery. [34,68] Some vaccines are specifically recommended to be given intradermally for optimal immune responses, eg rabies, hepatitis and cholera vaccines [31-33]. The intradermal delivery of vaccines may directly access the skin-dwelling antigen presenting cell compartment and facilitate *in vivo* targeting. Additionally, intradermal delivery allows the use of lower doses of certain vaccines while inducing the same or better immune response [31,34a-c]. Since this study was designed to evaluate the potential for improved immunogenicity of the existing vaccine candidate vCP205, we chose to administer as much of the vCP205 vaccine as feasible by the ID route. We are attempting to improve the immune response to the vaccine and will give a comparable dose using a reasonable number of injections. Intradermal vaccines are limited by the volume that can be delivered into the intradermal compartment, approximately 0.1-0.15 ml per injection. We plan to give 4 x 0.125 ml ID injections for each of the vaccinations which will deliver one-half of the IM dose (i.e. 0.5 ml ID vs. 1.0 ml IM).

Group III. ALVAC-HIV vCP205 has been previously tested in humans (intramuscular route) and found to be apparently safe and moderately immunogenic. This arm evaluates the response to vCP205 given in the usual IM route as an internal referent. Responses to this vaccine in humans are well characterized and will establish the external validity and generalizability of this study and provide a means of reference to the existing standard vaccine delivery system.

6.3 Assessment of Immunogenicity

6.3.1 Primary Measures of Immunogenicity--All Subjects

Immunogenicity: Description of the following primary measures of cellular immunogenicity

• Lymphocyte proliferation responses to specific vaccine component antigens (which includes gp160 MN/LAI protein from Aventis Pasteur and p24 LAI protein from MicroGeneSys) and to the control antigen KLH (for DC arm only).

• Cytotoxic T-lymphocyte (CTL) responses against vaccine component antigens expressed in a recombinant poxvirus vector system (expressing HIV env MN antigen, gag and pol LAI antigens) in standard bulk chromium release assays.
• ELISPOT analysis for gamma-interferon production from bulk PBMCs in response to vaccine antigen stimulation (or other relevant antigens as they become available) in an overnight culture system.
• Enumeration and characterization of antigen specific gamma-interferon production using flow cytometry

6.3.2 Ancillary Immunogenicity Studies

Measurement of breadth of antibody response by characterizing

-serum binding and neutralizing antibody to vaccine component antigens, including anti-ALVAC antibodies (ELISA)
-anti-KLH binding antibodies (ELISA) in dendritic cell arm only
-neutralizing antibody activity against primary isolates in vaccinee sera that display high level of neutralizing activity against the HIV-1 MN strain.

Measurement of breadth of cellular response by quantifying and characterizing

- modified bulk CTL assay using autologous dendritic cells for *in vitro* stimulation
- identification of selected cytokine/chemokine production from supernatants from LPA
- mapping of cross-reactive cellular epitopes by measuring lymphocyte proliferation and CTL activity against proteins and/or peptides from diverse HIV-1 subtypes.

6.4 Selection of Study Populations

The total study population consists of 36, healthy low-risk HIV-1 seronegative U.S. adult volunteers from the community and can include civilians or DOD dependents. Subjects will be 18 through 55 years of age (< 10% will be > 50 years old). Subjects should not be engaging in highest risk behavior for HIV (i.e., injection drug use or sex with HIV positive partner). Women must agree to practice effective contraception for 60 days prior to study entry and until 6 months after the fourth immunization. Subjects will be followed up for 6 months after the fourth immunization and will then have their study termination visit according to the protocol schedule of events. Volunteer recipients who experience HIV-1 infection during the trial or up to 18 months after the last immunization, will be offered enrollment in a study to evaluate the “natural history” of HIV-1 occurring in vaccine recipients, if eligible and referred to a local physician for follow-up health care.

6.4.1 Inclusion Criteria:

Volunteers are eligible for this study if they meet all the following criteria:

1. Legal residents of USA who are not at high-risk for HIV infection.
2. Age: 18 to 55.
3. For women, a valid negative serum pregnancy test prior to vaccination will be required, as well as verbal assurance that adequate birth control measures are applied 60 days prior to the first vaccination and for 6 months after the final vaccination.
4. Good health as determined by medical history, physical examination, and clinical judgment.
5. Clinical laboratory values at screening within the following normal ranges:
   - Hematocrit: Women: 35 % to 45 %; Men 36 % to 49 %
   - White cell count: 3,000 to 10,800 cells/mm^3

- Platelets: 150,000 to 400,000 per mm^3

6. Urinalysis for protein and blood: negative or trace. If either is ≥1+, repeat the UA. If evidence of blood or protein on repeat UA (microscopic and repeat dipstick) is confirmed, the volunteer is ineligible and will be appropriately referred for diagnosis and treatment.
7. Normal ALT (<1.5x institutional upper normal limit) and creatinine (0.1 - 1.6 mg/dL)
8. Negative serology for HIV infection (ELISA test), HIV RNA below detection limits of FDA approved viral load diagnostic assay.
9. Availability for at least 15 months of participation.
10. Successful completion of the Test of Understanding (maximum of 3 attempts to pass the test). Commitment for Trial Participation and signature of the approved consent form.
11.Successful EBV transformation of subjects B-cells obtained at the minus 90 to 30 day screening visit. If first attempt at EBV transformation is unsuccessful, a second attempt (blood draw) can be made. Subject is enrolled into study only if the EBV transformation is successful. A modified screening evaluation (that will include screening labs) will be repeated if greater than 12 weeks prior to date of enrollment. The EBV-transformation step is critically important because these transformed cells are required for the CTL assay, which allows the investigators to monitor the cellular immune response to the vaccine.

6.4.2 Exclusion Criteria:

Individuals will not be enrolled into the study if they meet at least one of the following criteria:

1. Acknowledge engaging in highest-risk behavior within 6 months of study entry: (e.g., active injecting drug use or having sexual intercourse with a known HIV-1 infected partner).
2. Have active tuberculosis or other systemic infectious process by review of systems and physical examination.
3. Have a history of significant cardiac arrythmias (ventricular tachycardia or ventricular fibrillation), seizure disorder, immunodeficiency, chronic illness, autoimmune disease**,** diabetes mellitus (Type I or II pre-existing diagnosis, or random glucose ≥ 200 or fasting plasma glucose ≥126- see diagnostic criteria in the report: Expert Committee on the Diagnosis and Classification of Diabetes Mellitus *Diabetes Care* Vol 25, Suppl 1, Jan 2002) or use of immunosuppressive medications.
4. Have evidence of psychiatric, medical and/or substance abuse problems during the past 6 months that the investigator believes would adversely affect the volunteer's ability to participate in the trial.
5. Have occupational or other responsibilities that would prevent completion of participation in the study.
6. Have received any live attenuated vaccine within 60 days of study entry.

NOTE: Medically indicated subunit or killed vaccines (e.g., Hepatitis A, Hepatitis B or rabies) are not exclusionary but should be given at least 2 weeks before, or 2 weeks after, HIV immunizations to avoid potential confusion of adverse reactions. However, if rabies vaccine is indicated in the post-exposure setting, it will take priority over the study vaccine.

7. Have used experimental therapeutic agents within 30 days of study entry.
8. Have received blood products or immunoglobulins in the past 3 months.
9. Have a history of anaphylaxis or other serious adverse reactions to vaccines.
10. Have previously received an HIV vaccine.
11. Are pregnant or breastfeeding.

NOTE: Women of child-bearing potential must be using effective contraception for at least 60 days prior to initial vaccination and for at least 6 months after the final vaccination.

12. Have chronic or active hepatitis B or Hepatitis C virus infection or active syphilis (positive RPR and FTA).
13. Have an immediate type hypersensitivity reaction to eggs, egg products or neomycin (used to prepare ALVAC vaccine) or gentamicin (used in preparation of the DC).

14. Are study site employees who are involved in the protocol and may have direct access to the immunogenicity results.
15. In the event that we are unable to generate autologous dendritic cells (DC) in sufficient quality or quantity from a volunteer who is randomized to the DC arm (Group I)-that volunteer will be excluded.

6.4.3 Removal of Subjects from Trial or Assessment

A subject may withdraw his/her consent to participate in the study at any time without prejudice. Additionally, the investigator may withdraw a subject if, in his/her clinical judgment, it is in the best interest of the subject or if the subject cannot comply with the protocol. Wherever possible, the tests and evaluations listed for the termination visit should be carried out if the subject refuses follow-up according to the protocol visit schedule. The sponsor should be notified of all study withdrawals within 24 hours. Subjects who withdraw from the study will be replaced only as long as the study is still open for enrollment. No replacement will be allowed when the enrollment is closed.

Obtaining Replacement Vial Assignment: If a subject withdraws from the study and is to be replaced, the principal investigator/study coordinator must contact DCAC to receive a replacement number. Replacement will only be permitted during the active enrollment phase. A fax or telephone message must be sent to DCAC providing the subject identification number of the withdrawn participant.

All subjects who receive at least one immunization will be included in the safety analysis. Only subjects who have received four injections and have had the Visit 11 blood draw (2 weeks post-fourth immunization) will be included in the final immunogenicity analysis, though immunogenicity data may be used in the analysis of earlier timepoints. If a subject misses an immunization visit (i.e., visit 4 or 7), no further immunizations will be performed, but the subject will continue to be followed according to the protocol visit schedule. If a subject misses a non-immunization visit (i.e., visit 3 or 5), immunization will continue as planned. A missed visit is defined as outside the allowed window for each visit. If a subject does not complete the immunization schedule secondary to a serious adverse event or toxicity, he or she will continue to be followed according to the protocol visit schedule, and, at a minimum, until the adverse event/toxicity is resolved and/or the cause is identified.

A genuine effort will be made to determine the reason(s) why a subject fails to return for the necessary visits. If the subject is unreachable by telephone, a registered letter, at the minimum, will be sent to the subject requesting contact with the clinic. This information will be recorded on the appropriate source document. For subjects who become pregnant before completing the vaccine series, no further immunizations will be given regardless of outcome. The subject will be followed for all remaining scheduled visits according to the schedule of procedures for safety evaluation. For all subjects who become pregnant while participating in the trial, a Pregnancy CRF will be completed. The site will maintain contact with pregnant subjects to obtain pregnancy outcome information for the Pregnancy Follow-up CRF (See Appendix 17.4).

6.5 Administration of Vaccines

6.5.1 Summary of Vaccines

Group I. Aventis Pasteur ALVAC-HIV transfected autologous DC (vCP205) /0.4 ml volume dose
-Each volunteer will have autologous DC prepared using GLP. DC will be infected with the vCP205 vaccine. The DC will be checked for sterility, identity and potency prior to release for vaccination.

-These vaccine-infected (vCP205 vial contents ~$10^{6.5}$ $TCID_{50}$ reconstituted in 1 ml volume, numbers of vials used will be determined by the numbers of available DC, 1 vial/2-3 x 10^6 DC) autologous dendritic cells are washed and resuspended in 0.4 ml sterile saline and 5% autologous plasma and injected subcutaneously within four hours (preferentially within two hours) of harvesting from culture. The anticipated range of numbers of DC to be reinfused is 1.5-6.0 x 10^6 cells.

The autologous DC product will be tracked throughout the study using unique patient identifiers (PIN), a unique laboratory DCDN (dendritic cell donor number) and bar coding, adhering to blood banking standards and GLP. Strict control will be maintained through multiple cross checks, the performance of a single "operation" (manipulation of cells in the laboratory) at any given time, and the use of careful documentation. Various techniques (tracking documents to be generated on each cell preparation for each donor) will be employed to ensure the autologous nature of the vaccine (Please see **Appendix A** for details of cell preparation and release tests).
-Samples will be sent for sterility testing by standard microbiologic methods 2 days prior to and on the day of vaccination. (sent out to referral diagnostic lab-Quest Diagnostics)
-evidence of bacterial contamination will be checked using the Gram stain method on the final product immediately prior to vaccination and
-endotoxin levels will be determined (using an LAL assay) on the final product prior to release for vaccination
-mycoplasma detection using PCR techniques will be performed two days prior to vaccination such that results will be available prior to vaccination and
-samples of the final product will be sent for mycoplasma culture immediately prior to vaccination.
Reasons to **hold** the product will be a positive Gram stain or positive 48hr. culture result, evidence of endotoxin, evidence of mycoplasma contamination or DC viability less than 70%. Identity must be completely tracked and verified with documentation. Other tests will be performed but results will not be a hold issue. These are detailed in **Appendix A**.

If a volunteer is justifiably vaccinated outside a window, all subsequent visits will be scheduled in reference to that most recent vaccination. For example, if the vaccination visit is 6 weeks late, the safety visit will still occur 2 weeks after that vaccination. All subsequent vaccination intervals will occur as originally planned in the protocol and consequently the schedule will just shift. Justifiable vaccinations given outside a window may be due to manufacturing or production issues or severe scheduling constraints outside the investigators' or volunteers' control. An important aspect of the scheduling for DC vaccinations is that the schedule is already quite constrained as we generally only give DC injections on Wednesdays or Thursdays. This is because of all the release testing that is required for a DC injection. Also, it takes one week to prepare a DC vaccine; compared to the other routes (IM or ID) which can be given anytime simply by taking the vaccine off the "shelf". The combination of a long preparation time and restricted vaccination days make out of window events more likely to occur in this arm. These out of window events due to manufacturing issues, etc are to be distinguished from the compliance ranges specified in the Section 6.4.3 Removal of Subject form Trial."

Group II. Aventis Pasteur ALVAC-HIV (vCP205) ~$10^{6.5}$ $TCID_{50}$ reconstituted in1 ml of sterile water.
- Recombinant Canarypox that expresses (*env/gag/pro*) HIV genes

Supplied as lyophilized product, reconstituted in 1.0 ml sterile water and given as 4 x 0.125 ml intradermal injections for a total dose of 0.5 ml. (Please note the ID dose is one-half the IM dose).

Group III. Aventis Pasteur ALVAC-HIV (vCP205) ~$10^{6.5}$ $TCID_{50}$ reconstituted in 1 ml of sterile water.
- Recombinant Canarypox that expresses (*env/gag/pro*) HIV genes

Supplied as lyophilized product, reconstituted in 1.0 ml sterile water and given as a single 1 ml intramuscular injection (for a total dose of 1.0 ml).

6.5.2 Identity of Investigational Products

There will be one vaccine used throughout the study, Aventis Pasteur ALVAC-HIV (vCP205). It will be suspended in sterile water or sterile saline depending on the route used for vaccine administration.

For the autologous vaccine-transfected DC route, the cells will be tracked throughout the study and handled in such a way to ensure identity. Only one set of donors' cells will be operated on at any time in the laboratory or clinic. Each donor will have their own shelf area within the incubator and be physically isolated from other donors' cells. A unique PIN, DCDN (dendritic cell donor number) and bar coding will be used to track each donors cells and ensure the autologous and safe reinjection of DC.

6.5.2.1 Vaccine Supplies

Component	Amount Per Dose
**ALVAC-HIV (vCP205) Lot #** TBD	~$10^{6.5}$ $TCID_{50}$/ml
10mM Tris HCl, pH 9	0.25 ml
lactoglutamate	0.25 ml
freeze–drying medium according to formula 65-1-2	0.50 ml

Storage: Store refrigerated at 2-8°C

6.5.2.2 Procedure for Reconstitution

ALVAC-HIV (vCP205) transfected DC

1). Autologous DC transfected with vCP205 vaccine (vCP205 vial contents ~$10^{6.5}$ $TCID_{50}$ reconstituted in 1 ml volume; numbers of vials used will be determined by the numbers of available DC, 1 vial/2-3 x 10^{6} DC) are washed free of the vaccine and resuspended in 0.4 ml sterile saline containing 5% autologous plasma.
2). Autologous vCP205- DC are infused into the superficial subcutaneous space in 2 x 0.2 ml aliquots within four hours of cell harvesting.

DC will be checked for quality, sterility, and identity prior to release into the volunteer. Limited phenotypic analysis (CD83, CD86, and CD14) as determined by flow cytometry of the matured DC will be performed prior to injection to confirm DC quality and maturation. Efficacy of transfection and gene expression will be determined on the day of injection but will not be a hold issue. Please see Appendix A for details.

ALVAC-HIV (vCP205) Intradermal ~$10^{6.5}$ $TCID_{50}$/vial

1). For the intradermal injection, add 1.0 ml of sterile water for injection

2). Shake mixture gently.
3). Withdraw 0.5 ml ALVAC-HIV (vCP205) and administer within 1 hour intradermally, 4 x 0.125 ml injections into the volar aspect of the arm.

Note: there will be 0.5 ml of vaccine (surplus) remaining in the vial after ID injection. Please see Section 6.5.2.4. for appropriate DISPOSITION.

ALVAC-HIV (vCP205) Intramuscular ~$10^{6.5}$ $TCID_{50}$/vial
1). For the intramuscular injection, add 1.0 ml sterile water for injection
2). Shake mixture gently.
3). Withdraw 1.0 ml ALVAC-HIV (vCP205), and administer within 1 hour, intramuscularly (IM), single injection into deltoid.

.

6.5.2.3 Method of Dosage Administration

ALVAC-HIV is kept at 2°-8°C.

A 5-part label is applied on the bag containing the syringe of ALVAC, placebo, or the DC, and it must mention:

Protocol #:
ALVAC
Subject #:
Immunization #:
Date: Time:
Use within 1 hour
Intramuscular injection in the deltoid, or Intradermal injection into volar aspect of arm, or subcutaneous injection into volar aspect of arm.
Caution: New drug limited by United States law to investigational use

From the 5-part tear-off labels applied to the bag containing the syringe(s):

1 part goes on the pharmacy book
1 part goes on the syringe
1 part goes onto the source document
1 part goes on the case report form
1 part stays on the bag

Syringe contents will not be noted on the CRF vaccine labels.

6.5.2.4 Disposition

Investigator or his/her designee is responsible for maintaining an accurate inventory and accountability record of vaccine supplied for this study. At the conclusion of vaccine administration, all vaccine supplies [including used, unused or partially used vials of ALVAC-HIV (vCP205)] must be returned to the VCRC. The division of Regulatory Affairs will return them to the manufacturer or destroy them. Partially used vials may not be administered to other subjects or be used for *in vitro* or animal model experimental studies.

6.5.2.5 Precautions to be Observed in Administering Study Vaccine

Study vaccine must not be administered to individuals with hypersensitivity to any component of the vaccine (refer to Section 6.4.2).

As with any parenteral vaccine, epinephrine, and corticosteroids must be available for immediate use should an immediate hypersensitivity reaction, such as anaphylaxis, occur. All study vaccines must be injected as described intradermally, intramuscularly or subcutaneously. **DO NOT** inject intravenously.

6.5.3 Method of Assigning Subjects to Study Groups

Subjects who meet initial admission criteria will be assigned personal identification numbers (PIN) and screening numbers at the first screening visit. After a volunteer is found to be eligible, the screening number will become the study number. This means that study numbers may be discontinuous. All three arms will be enrolled concurrently and subjects will be randomly assigned to either of the three arms. In Group I, 12 enrolled HIV seronegative adults will be given vCP205 transfected autologous dendritic cells pulsed with KLH (8) or KLH pulsed DC only (4). The KLH is used in the first vaccination only. In Groups II and III, 24 HIV seronegative volunteers will be randomized into 2 vaccine/placebo groups (an ID arm with 8 active agents/4 placebo and an intramuscular arm with 8 active agents/4 placebo). Within each group the subjects will be randomly assigned to active vaccine or placebo vaccine according to a list generated by DCAC.

6.5.4 Controls/Blinding

This trial is a prospective randomized, double-blinded (within each arm), placebo-controlled trial. The placebo controls are inactive substances (e.g. sterile saline) or KLH controls for each vaccination strategy as follows:
All arms use saline as the controls. In addition, the dendritic cell arm has a KLH control antigen that is added to all dendritic cells (active and placebo) for the first vaccination only.
A clinical pharmacist will prepare each vaccine dose (IM, ID) and deliver the doses to the research nurse for injection. The autologous-DC vaccines will be prepared in the Clinical DC laboratory and given to the research nurse for injection. Patients and investigative site personnel will be blinded within each group. Within each group the volume of injection will be consistent. The investigative site personnel, as well as WRAIR personnel involved in the monitoring or conduct of the trial, will be blinded to the study vaccine code. However, in case of an emergency situation occurring in one of the study volunteers, the code of this subject might be broken. The sponsors must be informed as soon as possible if the code is broken.

6.5.5 Prior and Concomitant Medications/Vaccines

The following medications should not be used while the subject is on the study:

1). Immunomodulatory agents or Immunosuppressive agents (i.e., systemic steroids, chemotherapy or others as determined by the PI).
2). Other experimental vaccines, except rabies, Hepatitis A, Hepatitis B (see exclusion criteria), within 60 days of immunization.
3). We ask that volunteers establish a 2 week window period around immunizations for other licensed, medically indicated vaccines (e.g. influenza).

6.5.6 Study Procedures

Pre-Study Screening Visits:

Visit 0, Day –120 to -60

1. Obtain informed consent for pre-trial screening.
2. Perform qualitative HCG assay (serum) for females.

(if positive, stop screening process)

3. Assign PIN and screening number.
4. Obtain and record medical history, including allergy history, with particular attention given to inclusion and exclusion criteria.
5. Perform physical examination, including weight, height, and oral temperature.
6. Provide pre-HIV test counseling.
7. Collect samples for HIV serology (1 ml red top).
8. Collect sample for CBC (3 ml EDTA lavender top).
9. Collect blood for EBV transformation of subject's B cells. If the first attempt is unsuccessful, a second blood draw will be done and all laboratory studies will be repeated.
10. Collect sample for RPR (syphilis), HbsAg, and Hep C and sample for chemistries to include Chem-7, LFTs, ALT and creatinine (8.3 ml, SST red top).
11. Collect urine for UA. If urine protein or blood is $\geq$1+, repeat the UA. If evidence of blood or proteinuria on repeat UA (microscopic and repeat dipstick) is confirmed, the subject is ineligible and will be appropriately referred for diagnosis and treatment.
12. Collect blood for baseline autoimmunity tests (ANA, RF and anti-thyroid antibodies), 2 ml red top.
13. Perform baseline extended risk assessment and provide counseling on avoidance of risk behavior.
14. Give subject a copy of vaccine informed consent to review at home.
15. Complete the Volunteer Registry Data Base Sheet

Visit 1, Day –60 to 0 (+/- 14 days): All volunteers

1. Test of understanding to be successfully completed
2. Enroll volunteer into the study
3. Perform interim history and physical and abbreviated risk assessment.
4. Consent explained/discussed, signed and pre-study leukopheresis performed in order to obtain cells for baseline immunomonitoring; and in those volunteers in the DC arm, as a cellular source for generation of their DC.
5. **If anticipated vaccination date (Visit 2) will occur more than 12 weeks from screen date, obtain a CBC, Chemistry, CK and UA.**

Visit 2, Day 0: First Vaccination

1. Review all screening data and inclusion/exclusion criteria to ensure eligibility.
2. Obtain subject signature on vaccine consent form. Provide a copy to the subject.
3. Perform serum pregnancy test for women before vaccination.

[Results must be known prior to immunization.]

4. Obtain interim history and physical, including vital signs and abbreviated risk assessment.
5. Perform risk behavior avoidance counseling and provide HIV test counseling.
6. Collect samples for HIV serology (1 ml red top).

7. Collect samples for immunogenicity (40 ml anticoagulated blood): LPA, neutralizing antibody, CTL, and ELISPOT. Separately and additionally, draw 3 ml of blood into a heparinized pediatric tube (no substitutions) PRIOR to immunization (whole blood ICC).
8. Administer vaccine injection(s) into the deltoid muscle (IM), or intradermally on the volar aspect of the arm (ID). The autologous DC are prepared from the pre-study leukopheresis. The DC are counted, checked for sterility, identity and potency and after being cleared for release, injected into the superficial subcutaneous space on the ventral inner aspect of the arm in two 0.2 ml aliquots. See details in **Appendix A** for specifics on dendritic cell release criteria, counts and maturity. Examine the injection sites for local reactions at 60 minutes post-injection and instruct subject in the evaluation of these local reactions; observe and instruct while the subject takes temperature (as guided for subsequent evaluations); enter findings on appropriate CRF.
9. Subjects will record daily symptoms for 7 days post-immunization (including the day of immunization), and will be instructed to contact the investigator if any untoward effects occur.

24-48 hour contact after the first immunization:

Subjects will be contacted by staff within 24 to 48 hours post-immunization (by telephone, by home visit, or by the other prearranged mechanism) to determine whether adverse effects have occurred. Subjects will be queried using a predetermined review of systems/reactogenicity worksheet. If the adverse effects experienced by the volunteer are deemed "moderate", he/she will be asked to return to clinic for evaluation by the investigator. Additional contacts will be arranged as indicated.

Visit 3: Day 14 (+/-4 days) after initial vaccination: Safety Evaluation

1. Collect diary, obtain interim history and physical.
2. Collect sample for CBC.
3. Collect sample for Chem-7, LFT's (Na, K, Cl, HCO3, Cr, BUN, Glc, AST, ALT, Total Bili, alk phos).
4. Collect urine for dipstick. If urine protein or blood is 1+ or greater, obtain a complete UA.

Visit 4: Day 28 (+/- 7 days) after initial vaccination:

Second vaccination

1. Obtain interim history and physical including vital signs and abbreviated risk assessment.
2. Perform serum pregnancy test for women before immunization.
   Results of pregnancy test must be known prior to vaccination.
3. Collect samples for immunogenicity (40 ml anticoagulated blood): LPA, neutralizing antibody, CTL and ELISPOT. Separately and additionally, draw 3 ml of blood into a heparinized pediatric tube (no substitutions) PRIOR to immunization (whole blood ICC).
4. Collect archival blood specimen (10 ml of anticoagulated blood).
5. Administer vaccine injection(s) into the left deltoid muscle (IM), or the volar aspect of the left arm (ID). The autologous DC are prepared from the pre-study leukopheresis. The DC are checked for sterility and identity and after being cleared for release, injected into the superficial subcutaneous space on the ventral inner aspect of the left arm in two 0.2 ml aliquots. Examine the injection sites for local reactions at 60 minutes post-injection for all injections and instruct subject in the evaluation of these local reactions; observe and instruct while the subject takes temperature (as guided for subsequent evaluations); enter findings on appropriate CRF.
6. Subjects will record daily symptoms for 7 days post-immunization (including the day of immunization), and will be instructed to contact the investigator if any untoward effects occur.

24-48 hour contact after the second immunization:

Subjects will be contacted by staff within 24 to 48 hours post-immunization (by telephone, by home visit, or by the other prearranged mechanism) to determine whether adverse effects have occurred. Subjects will be queried using a predetermined review of systems/reactogenicity worksheet. If the adverse effects experienced by the volunteer are deemed "moderate", he/she will be asked to return to clinic for evaluation by the investigator. Additional contacts will be arranged as indicated.

Visit 5: Day 42 after initial vaccination, 14 days (+/-4 days) after second vaccination: Safety Evaluation

1. Collect diary, obtain interim history and physical.
2. Collect sample for CBC.
3. Collect sample for Chem-7, LFT's (Na, K, Cl, HCO3, Cr, BUN, Glc, AST, ALT, Total Bili, alk phos).
4. Collect urine for dipstick. If urine protein or blood is 1+ or greater, obtain a complete UA.
5. Collect sample for cellular immunogenicity assays (40 ml of anticoagulated blood): LPA, CTL and ELISPOT. Separately and additionally, draw 3 ml of blood into a heparinized pediatric tube (no substitutions) PRIOR to immunization (whole blood ICC).
6. Collect archival blood specimen (10 ml of anticoagulated blood).

Visit 6: Day 60 after initial vaccination, 32 days (+/-7 days) after second vaccination

1. Obtain interim history including vital signs and abbreviated risk assessment.
2. Collect blood for autoimmunity tests (ANA, Rheumatoid factor, and anti-thyroid antibodies), 2 ml red top.
3. Collect samples for immunogenicity (40 ml anticoagulated blood): LPA, neutralizing antibody, CTL and ELISPOT.

Visit 7: Third Vaccination, Day 90 (+/- 14 days) after initial vaccination

1. Obtain interim history and physical including vital signs and abbreviated risk assessment.
2. Collect samples for HIV serology (1 ml red top).
3. Perform serum pregnancy test for women before immunization.
   Results of pregnancy test must be known prior to vaccination.
4. Administer vaccine injection(s) into the left deltoid muscle (IM), or the volar aspect of the left arm (ID). The autologous DC are prepared from the pre-study leukopheresis. The DC are checked for sterility and identity and after being cleared for release, injected into the superficial subcutaneous space on the ventral inner aspect of the left arm in two 0.2 ml aliquots. Examine the injection sites for local reactions at 60 minutes post-injection and instruct subject in the evaluation of these local reactions; observe and instruct while the subject takes temperature (as guided for subsequent evaluations); enter findings on appropriate CRF.
5. Subjects will record daily symptoms for 7 days post-immunization (including the day of immunization), and will be instructed to contact the investigator if any untoward effects occur.

24-48 hour contact after the third immunization:

Subjects will be contacted by staff within 24 to 48 hours post-immunization (by telephone, by home visit, or by the other prearranged mechanism) to determine whether adverse effects have occurred. Subjects will be queried using a predetermined review of systems/reactogenicity worksheet. If the adverse effects experienced by the volunteer are deemed "moderate", he/she will be asked to return to clinic for evaluation by the investigator. Additional contacts will be arranged as indicated.

Visit 8: Day 104 post-initial vaccination, 14 days (+/-4 days) post third vaccination:

Safety evaluation

1. Collect diary.
2. Obtain interim history and physical including vital signs, symptom assessment and examination of injection sites, including lymph nodes.
3. Collect sample for CBC.
4. Collect sample for Chem-7, LFT's (Na, K, Cl, HCO3, Cr, BUN, Glc, AST, ALT, Total Bili, alk phos).
5. Collect urine for dipstick. If urine protein or blood is 1+ or greater, obtain a complete UA.
6. Collect sample for cellular immunogenicity assays (40 ml of anticoagulated blood)-LPA, CTL, and ELISPOT. Separately and additionally, draw 3 ml of blood into a heparinized pediatric tube (no substitutions) PRIOR to immunization (whole blood ICC).
7. Collect archival blood specimen (10 ml of anticoagulated blood).

Visit 9: Day 120 post-initial vaccination, 30 days (+/-14 days) post third vaccination:

1.Obtain interim history and physical including vital signs.
2.Collect samples for immunogenicity (40 ml anticoagulated blood): LPA, neutralizing antibody, CTL and ELISPOT.

Visit 10: Day 180: Fourth and final vaccination, Day 180 (+/- 14 days) post-initial vaccination:

1. Obtain interim history and physical including vital signs and extended risk assessment.
2. Perform serum pregnancy test for women before immunization.
   Results of pregnancy test must be known prior to vaccination.
3. Administer vaccine injection(s) into the left deltoid muscle (IM), or the volar aspect of the left arm (ID). The autologous DC are prepared from the pre-study leukopheresis. The DC are checked for sterility and identity and after being cleared for release, injected into the superficial subcutaneous space on the ventral inner aspect of the left arm in two 0.2 ml aliquots. Examine the injection sites for local reactions at 60 minutes post-injection and instruct subject in the evaluation of these local reactions; observe and instruct while the subject takes temperature (as guided for subsequent evaluations); enter findings on appropriate CRF.
4. Subjects will record daily symptoms for 7 days post-immunization (including the day of immunization), and will be instructed to contact the investigator if any untoward effects occur.

24-48 hour contact after the fourth immunization:

Subjects will be contacted by staff within 24 to 48 hours post-immunization (by telephone, by home visit, or by the other prearranged mechanism) to determine whether adverse effects have occurred. Subjects will be queried using a predetermined review of systems/reactogenicity worksheet. If the adverse effects experienced by the volunteer are deemed "moderate", he/she will be asked to return to clinic for evaluation by the investigator. Additional contacts will be arranged as indicated.

Visit 11: Day 194 after initial immunization: Safety Evaluation- 14 days (+/- 4 days) after last vaccination. If unable to schedule the leukopheresis within this window, a separate safety visit (Visit 11a) will take precedence and be scheduled in the clinic. The leukopheresis visit (Visit 11b) will be scheduled within 2-6 weeks post final vaccination.

1. Collect diary.

2.Obtain interim history and physical including vital signs, symptoms, assessment, and examine injection sites, including lymph nodes.
3.Collect sample for CBC (3 ml EDTA).
4. Collect sample for Chem-7, LFT's (Na, K, Cl, HCO3, Cr, BUN, Glc, AST, ALT, Total Bili, alk phos), 3 ml red top.
5. Collect urine for dipstick. If urine protein or blood is 1+ or greater, obtain a complete UA.
6. Collect samples for final autoimmunity monitoring (ANA, RF and anti-thyroid antibodies), 2ml red top.
7. Collect sample for cellular immunogenicity assays (40 ml of anticoagulated blood)-LPA, neutralizing antibody, CTL, and ELISPOT. Separately and additionally, draw 3 ml of blood into a heparinized pediatric tube (no substitutions) PRIOR to immunization (whole blood ICC).
8. Perform HIV pretest counseling and obtain sample for HIV serology.
9. Review and sign consent for second leukopheresis.
10. Perform leukopheresis.

Visit 12: Day 264 after initial vaccination; 3 months (+/-14 days) after final vaccination

1. Obtain interim history and physical including vital signs.
2. Perform abbreviated risk assessment.
3. Collect samples for immunogenicity (40 ml anticoagulated blood): LPA, neutralizing antibody, CTL, and ELISPOT.
4. Collect archival blood specimen (10 ml of anticoagulated blood).

Study Termination Visit 13
Day 354 after initial vaccination; 6 months (+/-14 days) after final vaccination

1. Obtain complete history and physical exam including vital signs.
2. Perform extended risk assessment and provide risk behavior avoidance counseling.
3. Perform HIV serology test.
4. Collect samples for immunogenicity (40 ml anticoagulated blood): LPA, neutralizing antibody, CTL, and ELISPOT.
5. Collect archival blood specimen (10 ml of anticoagulated blood).
6. Perform qualitative HCG assay (serum) for females.

6.6 Study Variables and Their Measurement

6.6.1 Immunogenicity Variables

1. Cellular Immune Response to HIV-1 Antigens
   a. Cells for the assessment of lymphocyte proliferation to HIV gp160 MN and gag IIIB protein will be collected on days 0, 14, 28, 42, 60, 104, 120, 194, 264, and 354.
   b. Cells for the assessment of CTL activity and ELISPOT assay for interferon-g will be collected days 0, 14, 28, 42, 60, 104, 120, 194, 264, and 354.
   c. Cells for the enumeration and identification of antigen specific interferon-g production using flow cytometry will be collected on Day -10, 28, 120, 194, 264 and 354.

2. Humoral immune responses to HIV
   a. Antibody responses to homologous HIV-1 MN, gp120, and p24III-B and other non-hologous HIV-1 env antigens and peptides will be evaluated using plasma collected on days 0, 28, 60, 120, 194, 264, and 354.

b. Neutralizing antibody to HIV-1 MN strain, selected primary isolates, and other TCLA strains will be performed by the Immunology Laboratory at WRAIR, Rockville, MD.

3. Ancillary Studies

Human specimens collected under this protocol may be used to address other relevant questions regarding HIV-1 immunity if cells are available and CTL or neutralizations are elicited. Specifically, cells may be used to compare pre- and post-immune responses, compare assay methods of determination of cytotoxicity, characterize cytokine production, detect evidence of intracellular vaccine or cellular activation as a result of exposure to vaccine, or check for expression of vaccine components on the surface of autologous DC exposed to vCP205. Additionally, peripheral blood cells will be studied for evidence of sensitization to the vaccine (anti-ALVAC antibodies) and for evidence of primary cellular and humoral immunity against the test antigen KLH. Most methods described herein are standard immunologic techniques, however should new techniques become available to delineate HIV-1 immunity, these may be used as well. Aventis Pasteur, the US Army Medical Research and Material Command, and Rockefeller University will be informed of and consulted regarding these ancillary studies. These results will be discussed by the aforementioned parties prior to public disclosure.

6.6.1.1 Specimen Handling and Processing

1. Venipuncture will be done according to institutional guidelines.

2. Samples will be collected in the appropriate tubes as outlined in the attached table; substitution of tube types may be made as long as these are appropriate and do not interfere with the performance of the studies in question.

3. All biological samples to be analyzed by the clinical laboratory will be collected and processed according to institutional guidelines.

4. All samples sent to the Specimen Processing Center will be labeled with the following information:
Study number
Subject number: Subject initials:
Study day: Visit number:
Date
Type of sample (serum, plasma, blood etc)

8901 Samples that will be analyzed after freezing should be prepared, labeled, and stored at the appropriate temperature. Specimens that are shipped will be correctly prepared, labeled, and kept at the correct temperature; all necessary shipping licenses/permits will be obtained.

6.6.1.2 Sample collection

Laboratory study	Type	Storage	Tube*	Amount
Hematology	blood	fresh	EDTA	3 ml
Chemistry	serum	fresh	red	3 ml
HIV Western blot	serum	fresh/frozen	red	1 ml

Serology	serum	fresh/frozen	red	1 ml
gp120/41 antibody	ser/plas	frozen	heparin	10 ml
Lymphocyte proliferation	blood	fresh/frozen	heparin	10 ml/8 ml
Cytotoxicity assay	blood	fresh/frozen	heparin	10 ml/8 ml

*Equivalent, appropriate substitutions to tube types are allowed

6.6.2 Safety Variables

6.6.2.1 Post-immunization Events Monitored as Possible Reactions to Vaccine

Selected local and systemic adverse events are routinely monitored in vaccine clinical trials as indicators of vaccine reactogenicity. It is recognized that each of these events, and particularly those of a systemic nature, may under some circumstances, in any individual subject, have a cause that is unrelated to study vaccines. However, as a matter of convenience and in accordance with common clinical practice, all such events occurring within 7 days of immunization (including the day of immunization) are herein termed "*post-immunization reactions*".

Post-immunization Reactions Occurring Immediately After Immunization

Following each vaccine administration, subjects will be observed in the clinic for 60 minutes. Study personnel will then evaluate the subject for any signs or symptoms of local or systemic reactions. These will be noted in the case report form. Possible local reactions that should be recorded include erythema and induration (measured using the ruler indicated on the diary card that will subsequently be provided to the subject), and pain/tenderness, or warmth at the injection site. Specific symptoms to be noted include fever (described in Toxicity Grading Scale, p. 64), fatigue, headache, myalgia, arthralgia, chills, nausea/vomiting, shortness of breath, chest pains, itching and rashes. For each immunization, oral temperature will be recorded prior to immunization and 60 minutes after immunization.

Local Reactions Within 7 Days Post-immunization (Study Days 0 to 6)

The subject will be asked to note occurrences of erythema and induration (measured using the ruler included on the diary card), and pain/tenderness, or warmth at the injection sites daily for 7 days. These occurrences should be recorded on the diary card provided to serve as a reminder to the subject for the next clinic visit. A copy of the diary card will be kept by the study site.

Systemic Reactions Within 7 Days Post-immunization (Study Days 0 to 6)

The subject will also be asked to note occurrences of fever (>38^0C), fatigue, headache, myalgia, arthralgia, chills, nausea/vomiting, itching and rashes daily for 7 days and record these occurrences on the diary card mentioned previously. Oral temperature should be recorded by the subject 6 hours after immunization, and then daily for the next 7 days (for a total of 7 days of observation). If a medication is taken for treatment of fever, the temperature should be retaken and recorded 4 hours after the antipyretic dose to see if further treatment is necessary.

Post-immunization Contact

Between 24 and 48 hours after each immunization, the subject will be contacted by the investigator, study nurse, or research assistant to obtain local and systemic reaction data and to determine the subject's clinical status.

Instructions to Subjects Regarding Unusual or Severe Signs or Symptoms

Subject will be instructed to call the specified study personnel immediately if any unusual or severe sign or symptom appears after immunization. These subjects should be seen in the clinic at the time of maximal symptoms, if possible, and will be followed up clinically until resolution of symptoms.

6.6.2.2 Stopping Criteria

A severe adverse event or grade III/IV toxicity which is probably or definitely related to vaccination will result in discontinuation of vaccination for the individual. The volunteer will be asked to continue the follow-up schedule for the duration of the study.

Two severe adverse events or grade III/IV toxicity which are probably or definitely related to vaccination will result in discontinuation of the study arm. The volunteers will be asked to continue the follow-up schedule for the duration of the study.

Discontinuation of a study arm will prompt an immediate analysis of study safety data for all arms, a review of available immunogenicity data and consideration of modifying the design of the discontinued study arm (i.e.dose reduction) to minimize the risks to participants in the modified arm. New volunteers would be recruited for the modified study arm.

6.6.2.3 Other Adverse Events

An adverse event is any undesired, noxious or pathological change in a patient or subject as indicated by physical signs, symptoms, and/or laboratory changes that occurs following administration of one of the vaccines, whether or not considered vaccine related. This definition includes intercurrent illnesses or injuries, and exacerbation of pre-existing conditions. The following information will be included in IND adverse event reports: Subject identification number and initials; investigator's name and name of MTF; subject's date of birth, gender, and ethnicity; test article and dates of administration; signs/symptoms and severity; date of onset; date of resolution or death; relationship to the study drug; action taken; concomitant medication(s) including dose, route, and duration of treatment; date of last dose; and HSSRB protocol Log No A-9906.

Local reactions to immunization and selected systemic events will be solicited via subject interview and will be recorded on the post-injection reaction form. Reactions occurring within 7 days post-immunization will be recorded on a reactogenicity form. All other adverse events will be recorded on the adverse event form. All adverse events occurring up to 30 days after the fourth injections will be collected. After that, i.e., after visit 12 at week 37, only adverse events that are serious and/or necessitate a physician’s visit or a prescribed medication will be collected. The subject should be followed carefully until the condition is resolved and/or the cause is identified. Any medication or other therapeutic measure taken to relieve symptoms of the medical problem must be recorded on the appropriate case report form page(s) in addition to the outcome of the adverse event.

Where a diagnosis is possible, it is preferable to report this rather than a series of terms relating to the diagnosis. When reporting a syndrome, indicate the associated signs and symptoms parenthetically following the syndrome rather than as separate events.

The *severity of events* reported on the adverse event form will be determined by the investigator, based on the Toxicity Grading Scale (Appendix 17.6) or if the event is not listed the event will be graded following these guidelines:

Mild (Grade 1):	Transient or mild discomfort. No limitation in normal daily activity.
Moderate (Grade 2):	Some limitation in normal daily activity.
Severe (Grade 3):	Unable to perform normal daily activity.
Serious (Grade 4):	Life threatening
Death (Grade 5):	

The *relationship of immunization* to adverse event (AE) will also be determined by the investigator, based on the following definitions:

Not Related:
AE obviously explained by another cause; OR
The time of occurrence of AE is not reasonably related to vaccination.

Remotely Related:
AE more likely explained by causes other than vaccination.

Possibly Related:
Vaccine administration and AE occurrence reasonably related in time; AND
AE explained equally well by causes other than vaccination.

Probably Related:
Vaccine administration and AE occurrence reasonably related in time; AND
AE more likely explained by vaccination than by other mechanisms.

Definitely Related:
Vaccine administration and AE occurrence reasonably related in time; AND
Vaccination most likely explains the AE; AND
AE is consistent with pattern of vaccine-related events.

The definition of a serious adverse event is as follows:

A ***serious*** adverse event is defined as any untoward medical occurrence that:
-Results in death.
-Is life threatening (i.e., the patient was, in the opinion of the Investigator, at immediate risk of death from the event as it occurred).
-Requires or prolongs inpatient hospitalization (elective hospitalization excluded).
-Results in persistent or significant disability/incapacity, (i.e., the event causes a substantial disruption of a person's ability to conduct normal life functions).
-Is congenital anomaly/birth defect of a pregnancy arising while on study
-Is an important and significant medical event that, based upon appropriate medical judgment, may jeopardize the patient or subject and may require medical or surgical intervention to prevent one of the other outcomes defining serious. Written follow up information will be provided, when available, for all serious and unexpected adverse events in addition to the initial reports.

All unexpected adverse events (AE) will be reported to the WRAIR HURC and the HSRRB within 7 days. All deaths and any other serious adverse event will be reported to the FDA and the WRAIR

HURC and the HSRRB within 24 hours regardless of the perceived relationship with the vaccine. All other AE and SAE will be reported in accordance with all applicable regulations. Reports are made by telephoning the Secretary of the IRB and one of the following:

Mary A. Marovich, MD, DTMH
Dept of Vaccine Research
WRAIR
13 Taft Court, Suite 200
Rockville, MD 20850
w (301) 251-8337
fax (301) 762-4177
h (301) 469-4980

Beryl Wessner, PharmD
Chief Regulatory Affairs
Henry M. Jackson Foundation
One Taft Court, Suite 250
Rockville, MD 20850
w (301) 251-5037
fax (301) 294-1898
h (301) 654-4635

Telephonic reporting must be followed by a written report within 24 hours to the IRB and to the following study investigators/team members:

Mary A. Marovich, MD, DTMH
WRAIR
13 Taft Court, Suite 200
Rockville, MD 20850
w (301) 251-8337
fax (301) 762-4177
h (301) 469-4980

Beryl Wessner, PharmD
Henry M. Jackson Foundation
One Taft Court, Suite 250
Rockville, MD 20850
w (301) 251-5037
fax (301) 294-1898
h (301) 654-4635

Merlin Robb, MD
U.S. Military HIV Research Program
1600 E.Gude Drive
Rockville, MD 20850
w (301) 251-8302
fax (301) 762-4177
h (301) 236-9475

Sanjay Gurunathan, MD
Aventis Pasteur
1 Discovery Drive
Swiftwater, PA. 18370
USA
tel: (570) 839-6185
fax: (570) 839-0934

Further immunizations should not be given to any subjects who have experienced a serious adverse event, whether related to vaccine or not, until written approval is obtained from the Principal Investigator or their designee.

6.6.2.4 Diagnostic Algorithm to Differentiate Immunogenicity from HIV Infection:

Diagnostic HIV testing will be carried out using an algorithm as described below. Information to the clinical staff at the vaccine trial site will not include the results of specific tests, but will rather state only "infected" or "not infected". This system will allow timely HIV testing without compromising the double-blind nature of the trial.

An FDA-approved HIV-1 ELISA assay will be performed five times throughout the course of the protocol (days -90 to -30, 0, 90, 194, and 354). If the ELISA is reactive, an FDA-approved HIV Western blot will be performed. If the Western blot is positive, HIV molecular diagnostics (Roche Amplicor) will be performed.

If the molecular diagnostics assay is positive, the person is suspected of being HIV positive and called back within 2 weeks for repeat testing. A verification blood specimen is obtained for HIV RNA. If repeat Amplicor tests are positive (both positive), then the person is considered HIV positive.
If repeat tests are discordant, the person is called back and repeat testing is done until a definitive diagnosis is made (either the person is uninfected or infected).

6.6.2.5 Management of Volunteers with HIV-1 Infection During the Trial

1. If a volunteer is found to be HIV-infected during the trial, he/she will be excluded from further immunizations in the trial.

2. All vaccine recipients will also be offered diagnostic HIV testing and counseling 12 and 18 months after the last immunization (See HIV Testing Algorithm, Section 6.6.2.4).

3. Trial participants who are found to be infected with HIV-1 during the trial will be referred to a local physician and a designated clinic that specializes in long-term HIV care and treatment.

4. The staff of the designated clinic will be provided with the test results upon which the diagnosis of HIV infection is based. The clinic will also be provided with CD4 T cell count and HIV viral RNA quantitation (viral load).

5. Any trial participant who received vaccine and becomes HIV infected during the period of immunization, and up to 18 months post last immunization, will be offered enrollment, if eligible, in a protocol studying the natural history of HIV infection.

6.7 Procedural Flow Chart

6.8 Volunteer's Vaccine Schedule

6.9 Statistical Methods and Determination of Sample Size

6.9.1 Statistical Plan: Design and Sample Size Calculation

The data will be analyzed by DCAC. Any data analyses carried out independently by investigators should be submitted to WRAIR before publication or presentation.

The trial as designed has 3 groups:
Group I: Subcutaneous reinjection of *ex vivo* generated vCP205 transfected autologous dendritic cells
Groups II and III: Intradermal or intramuscular injection of vCP205

The primary safety measurements include data from the clinical laboratory tests, observed immunization reactions (local and systemic) and adverse events. The primary immunogenicity data focus on cellular responses which will be assayed by both blastogenesis (lymphocyte proliferation) and cell mediated cytotoxicity.

This study is intended as a proof-of-concept to test the safety of the dendritic cell and ID delivery routes. It is not a definitive hypothesis testing study aimed at evaluating the relative immunogenicities (as measured by CTL response) of the IM, ID and DC arms. Despite this, we were able to determine the power with which differences between the DC arm and the better of the IM or ID arms could be detected. Assuming 100% (8 of 8) CTL response in the DC arm and 38% (3 of 8) response in the IM or ID arm, we would have approximately 70% power to detect a true difference in CTL response between the two arms if one actually exists. Additional information provided by immunomonitoring using ELISPOTs and intracellular flow cytometry (for IFN-gamma production) and blastogenesis assays (lymphocyte proliferation), we believe, will allow the study to determine if DC or ID routes are substantially better than the IM route.

All subjects receiving at least one injection of vaccine will be included in the safety and tolerability analyses. All data exclusions, including premature terminations, will be detailed and tabulated according to vaccine group. Data listings will include all subjects.

Demographic data obtained during the baseline visit will be listed for each subject. Summary statistics will be tabulated for each vaccine group. Subjects will be assessed for comparability at baseline. Descriptive statistics will be presented for continuous variables (age, height, weight). Frequency counts and percents will be presented for categorical variables (age, sex, and race).

6.9.2 Safety

All subjects receiving an injection of vaccine or placebo will be included in the safety and tolerability analyses.

6.9.2.1 Postimmunization Reactions (Local and Systemic)

The maximal severity of local and systemic postimmunization reactions occurring in the seven days following each immunization and any immunization will be tabulated by group. Local reactions tabulated will include: injection site pain, erythema, induration, and temperature. Systemic reactions reported within seven days postimmunization will include: chills, nausea, malaise, myalgia, arthralgia, headache, rash and fever (as described in Toxicity Grading Scale, p. 58). Additionally, pain on injection will be noted. Frequencies and percentages of subjects experiencing each reaction will be presented for each symptom severity. If a reaction occurs more than once for a subject, the reaction will be classified according to the highest occurring severity and closest vaccine relationship.

Summary tables showing the occurrence of any local or systemic reaction overall and at each time point will also be presented.

6.9.2.2 Other Adverse Events

The original terms used by investigators on the CRF will be translated to COSTART terms and grouped into body system and presented as frequency tables. For each and any immunization, the number and percent of subjects with adverse events will be tabulated, by group and by site. When adverse events occur more than once, the maximal severity and relationship to vaccine will be counted. Three summaries will be generated: (1) Serious adverse events (SAEs); (2) adverse events that are definitely, probably or possibly related to vaccination, and (3) adverse events that are remotely or not related to vaccination. Data listings of all adverse events will be provided by subject, as will subset listings summarizing subjects withdrawn from the study because of an adverse event.

6.9.2.3 Clinical Laboratory

Listings and descriptive statistics will be generated for all laboratory parameters that are abnormal, by group and site. Data listings will be provided by subject summarizing all abnormal values, by parameter (WBC count, hemoglobin, platelet count, ALT, creatinine, U/A).

6.9.3 Immunogenicity

Lymphocyte proliferation will be described as a stimulation index (LSI = CPM of test antigen/Medium Control). For each group geometric mean LSI, median minimal and maximal LSI, as associated 95% confidence intervals will be calculated. The primary timepoint of interest for antibody responses will be one month post immunization #4; cellular responses will be measured as described in section 6.7. The following definitions establish discrete categorical data (response present vs absent) for significance testing:

Cellular:A positive lymphoproliferative response is present if an **LSI** ≥ 5 to an HIV-specific vaccine component develops at 2 timepoints post vaccination.

A **CTL** response is present if HIV-antigen specific lysis at two effector: target (E:T) ratios is > 10%. The response is CD8+ T cell mediated if, on subsequent testing (depletion assay), depletion of CD8+ T cells removes 50% of the HIV antigen specific lytic activity, while removal of CD4+ cells maintain at least 5% HIV specific lysis. Likewise, The response is CD4+ T cell mediated if, on subsequent testing, depletion of CD4+ T cells removes 50% of the specific lytic activity, while removal of CD8+ T cells maintains at least 5% specific lysis.

Cumulative % bulk CTL response is calculated as follows: number of participants with a CTL response to any vaccine component on any study day/total # of participants X 100 (for vaccine and placebo groups). Cumulative % CD8+ T cell mediated CTL response is calculated as follows: number of participants with a CD8+ T cell specific CTL response to any vaccine component on any study day/total # of participants X 100 (for vaccine and placebo groups). CTL responses will be categorized for the purpose of enumeration as: 1) high response, occurring on 5 or more occasions in an individual during the study, 2) medium response, occurring on 3-4 occasions and 3) low response, occurring on 1 or 2 occasions.

ELISPOT assay:
The number of cytokine (IFN-g or other) spot forming cells (SFC)/10^6 PBMC will be assessed to HIV vaccine components. Responses are calculated by subtracting the background SFC (in response to media alone) from the # of SFC to HIV vaccine components.
A positive **ELISPOT** response is defined by an absolute number of SFC
> 20 SFC/10^6 PBMC and SFC to HIV vaccine components at least twice those of background.

Humoral: A positive neutralization response is defined as >50% reduction in HIV gag antigen (vaccine related HIV strains vs normal human serum) in a continuous T cell line based neutralization assay

For each group, least squares geometric mean titer (GMTs), associated 95% confidence intervals, and median, minimal and maximal titer will be calculated to MN, LAI, gp120/160 antigens, by ELISA. Neutralizing antibody titer will be similarly measured to TCLA viruses and to select primary isolates.

Chi-square and Fisher's exact tests will be employed. All tests will be conducted as two-sided at the 0.05 significance level. Statistically significant results will be annotated by their degree (*p<0.05;**p<0.01;***p<0.001).

7. COMPENSATION, INSURANCE, INDEMNITY

7.1 Information on compensation, insurance and indemnity

Information on compensation, insurance and indemnity will be supplied to the investigator in the Clinical Study Agreement. Volunteers will be compensated for the last screening visit and for each scheduled vaccine visit according to the following schedule (not to exceed, unless receipt is presented): for each blood draw the volunteers will receive $50.00. For each leukopheresis the donor will receive $300.00. This means that each volunteer in the trial may be compensated up to $1250.00.

7.2 Advocacy

All volunteers will receive an individual identification card indicating their status as a vaccine trial participant. All volunteers will be counseled periodically regarding the potential for testing positive on routine screening tests for HIV-1 as a consequence of participation in this trial and receiving this vaccine product. All volunteers will be offered further confirmatory testing and certification as to the nature of their vaccine trial participation whenever needed to address complications arising at home, at work or in the community which could arise from routine screening for HIV-1.

8. VACCINE ACCOUNTABILITY

The investigator must maintain accurate records of dates and quantities, and lots of product(s) received, to whom dispensed (subject-by-subject accounting), and accounts of any product accidentally or deliberately destroyed. The investigator must retain all unused or expired product until the study monitor has confirmed accountability data.

At the conclusion of vaccine administration, all vaccine supplies (including used and unused vials) will be disposed of according to the procedure agreed upon. An overall summary of all vaccine supplies received, used and returned must be prepared at the conclusion of the study.

9. LABORATORY REQUIREMENTS

Clinical laboratory evaluations for safety will be performed at various time points during the trial.

10. CASE REPORT FORMS

Case report forms (CRF) will be provided for each subject. Correction to data on CRFs may be made only by putting a line through the incorrect data and writing the correct values, allowing the original text to remain legible. Each correction should be initialed and dated by the person making the change. If corrections are made after review and signature by the investigator, he/she must be made aware of the changes and document this awareness.

It is the policy of WRAIR that the study data must be verifiable to the source data, which necessitates access to all original recordings, laboratory reports, and subjects' records. The investigator must therefore agree to allow access to subjects' records, and source data must be made available for all study data.

11. STUDY MONITORING AND COMPLIANCE

All aspects of the study will be carefully monitored by the sponsor or authorized representatives of the sponsor, with respect to current good clinical practices and standard operating procedures for compliance with applicable government regulations. These individuals will have access, both during the trial and after trial completion, to review and audit all records necessary to ensure integrity of the data, and will periodically review progress of the study with the principal investigator.

Every attempt must be made to follow the protocol and to obtain and record all data requested for each subject at the specified times. However, ethical reasons may warrant the failure to obtain and record certain data, or to record data at the times specified. If this becomes necessary, the reasons for such must be clearly documented on the case report form.

12. PROTOCOL DEVIATION REPORTING

Emergent deviations or amendments may be initiated without prior Sponsor or IRB/ERC approval, only in cases where the change(s) is /are necessary to eliminate an immediate apparent hazard.
A protocol deviation is defined as an isolated occurrence involving a procedure that did not follow the study protocol, or study specific procedures.

The timeline for reporting protocol deviations to the Office of Research Management (ORM) WRAIR HURC and, if an IND study, Regulatory Compliance and Quality/USAMRMC HSRRB is determined by the categorization of the deviation: (1) emergent/significant or (2) non emergent/minor. Unanticipated problems should be reported in the appropriate timeframe according to the seriousness of the event as an SAE, a significant deviation, or a minor deviation.

Emergent/Significant deviations are departures from protocol that have a significant impact on the welfare or safety of a volunteer or on the integrity of the study data. Examples: administering the wrong test article to a volunteer; failure to obtain a scheduled blood draw for multiple participants. Significant deviations that occur in minimal risk and greater than minimal risk protocol will be reported to the sponsor (as required) and through the Office of Research Management (ORM) and WRAIR Human Use Review Committee, and if a greater than minimal risk (GTMR) protocol, to the Office of Research Protections, USAMRMC, immediately by phone and in writing within 5 business days of becoming aware of the deviation. Non Emergent/Minor deviations are routine departures that typically involve a volunteer's failure to comply with the protocol. Examples: missing scheduled visits; failing to return diary cards.

Minor deviations that occur in minimal risk and greater than minimal risk protocols will be reported to the sponsor (as required) and the Office of Research Management/HURC in a summary report with the annual continuing review report. A cumulative deviation report will be submitted to the ORM with each protocol continuing review report or with the final report, whichever comes first.

13. RETENTION OF RECORDS

Because data from clinical trials sponsored by the Office of the Surgeon General, Department of the Army (OTSG) may be used to support regulatory filings in several countries throughout the world, the policy concerning record retention reflects the most stringent current guidelines (those of the Committee for Proprietary Medicinal Products, or CPMP, in Europe). To comply with the CPMP guidelines, WRAIR requests that the investigator arrange for the retention of case report forms, source records, and other supporting documentation for a minimum of 15 years.

14. USE OF INFORMATION AND PUBLICATION

The investigator understands that the information generated in this study will be used by the sponsor in connection with the development of the product and therefore may be disclosed to government agencies in various countries. To allow for the use of information derived from the study, it is understood that the investigator is obliged to provide the sponsor with complete test results, all study data, and access to all study records.

WRAIR recognizes the importance of communicating medical study data and therefore encourages their publication in reputable scientific journals and at seminars or conferences.

Any results of medical investigations with Aventis Pasteur products and/or publication/lecture/manuscripts based thereon, shall be exchanged and discussed by the investigator, the manufacturer's representative(s), and the U.S. Army Medical Research and Materiel Command 30 days prior to submission for publication or presentation.

Results from investigations shall not be made available to any third party by the investigating team outside the publication procedure as outlined previously. WRAIR will not quote from publications by investigators in its scientific information and/or promotional material without full acknowledgment of the source (i.e., author and reference).

15. PROTOCOL AMENDMENTS

Only the sponsor may modify the protocol. Amendments to the protocol will be made only after consultation and agreement between sponsor and investigator. The only exception is where the investigator considers that a subject's safety is compromised without immediate action. In these circumstances, immediate approval of the chairman of the HSRRB and IRB must be sought, and the investigator should inform the sponsor and the full boards within 5 working days after the emergency occurred. All amendments that have an impact on subject risk or the study objectives, or require revision of the informed consent document, must receive approval from the HSRRB and all IRBs prior to their implementation. Minimal risk ammendments must be reviewed and approved prior to implementation.

16. REFERENCES

1. UNAIDS/WHO. AIDS epidemicupdate: December 1999.
2. Quinn TC. Global burden of the HIV pandemic. *Lancet* 348:99-106 (1996).
3. Steinman, R.M. & Witmer, M.D. Lymphoid dendritic cells are potent stimulators of the primary mixed leukocyte reaction in mice. *Proc. Natl. Acad. Sci*. USA 75, 5132-5136 (1978).
4. Inaba, K., Steinman, R.M., Van Voorhis, W.C. & Muramatsu, S. Dendritic cells are critical accessory cells for thymus-dependent antibody responses in mouse and man. *Proc. Natl. Acad. Sci*. USA 80, 6041-6045 (1983).
5. Inaba, K. & Steinman, R.M. Protein-specific helper T lymphocyte formation initiated by dendritic cells. *Science* 229, 475-479 (1985).
6. Inaba, K., Young, J.W. & Steinman, R.M. Direct activation of CD8+ cytotoxic T lymphocytes by dendritic cells. *J. Exp. Med*. 166, 182-194 (1987).
7. Bhardwaj, N., Friedman, S.M., Cole, B.C. & Nisanian, A.J. Dendritic cells are potent antigen-presenting cells for microbial superantigens. *J. Exp. Med.* 175, 267-273 (1992).
8. Seder, R.A., Paul, W.E., Davis, M.M. & Fazekas de St.Groth, B. The presence of interleukin 4 during in vitro priming determines the lymphokine-producing potential of CD4+ T cells from T cell receptor transgenic mice. *J. Exp. Med*. 176, 1091-1098 (1992).
9. Croft, M., Duncan, D.D. & Swain, S.L. Response of naive antigen-specific CD4+ T cells in vitro: characteristics and antigen-presenting cell requirements. *J. Exp. Med*. 176, 1431-1437 (1992).
10. Sallusto, F. & Lanzavecchia, A. Efficient presentation of soluble antigen by cultured human dendritic cells is maintained by granulocyte/macrophage colony-stimulating factor plus interleukin 4 and downregulated by tumor necrosis factor . *J. Exp. Med*. 179, 1109-1118 (1994).
11. Lechler, R.I.& Batchelor, J.R. Restoration of immunogenicity to passenger cell-depleted kidney allografts by the addition of donor strain dendritic cells. *J. Exp. Med*. 155, 31-41 (1982).
12. Inaba, K., Metlay, J.P., Crowley, M.T. & Steinman, R.M. Dendritic cells pulsed with protein antigens in vitro can prime antigen-specific, MHC-restricted T cells in situ. *J. Exp. Med*. 172, 631-640 (1990).
13. Liu, L.M.& MacPherson, G.G. Antigen acquisition by dendritic cells: Intestinal dendritic cells acquire antigen administered orally and can prime naive T cells in vivo. *J. Exp. Med*. 177, 1299-1307 (1993).
14. Sornasse, T., et al. Antigen-pulsed dendritic cells can efficiently induce an antibody response in vivo. *J. Exp. Med*. 175, 15-21 (1992).
15. Havenith, C.E.G., et al. T cell priming in situ by intratracheally instilled antigen-pulsed dendritic cells. *Am. J. Resp. Cell Mol. Biol*. 8, 319-324 (1993).
16. Flamand, V., et al. Murine dendritic cells pulsed in vitro with tumor antigen induce tumor resistance in vivo. *Eur. J. Immunol*. 24, 605-610 (1994).
17. Porgador, A. & Gilboa, E. Bone marrow-generated dendritic cells pulsed with a class I-restricted peptide are potent inducers of cytotoxic T lymphocytes. *J. Exp. Med*. 182, 255-260 (1995).
18. Zitvogel, L., et al. Therapy of murine tumors with tumor peptide pulsed dendritic cells: Dependence on T-cells, B7 costimulation, and Th1-associated cytokines. *J. Exp. Med*. 183, 87-97 (1996).
19. Celluzzi, C.M., Mayordomo, J.I., Storkus, W.J., Lotze, M.T. & Falo Jr., L.D. Peptide-pulsed dendritic cells induce antigen-specific, CTL-mediated protective tumor immunity. *J. Exp. Med*. 183, 283-287 (1996).
20. Paglia, P., Chiodoni, C., Rodolfo, M. & Colombo, M.P. Murine dendritic cells loaded in vitro with soluble protein prime CTL against tumor antigen in vivo. *J. Exp. Med*. 183, 317-322 (1996).
21. Mayordomo, J.I., et al. Bone marrow-derived dendritic cells pulsed with synthetic tumour peptides elicit protective and therapeutic antitumour immunity. *Nat Med*. 1, 1297-1302 (1995).

22. Boczkowski, D., Nair, S.K., Snyder, D. & Gilboa, E. Dendritic cells pulsed with RNA are potent antigen-presenting cells in vitro and in vivo. *J. Exp. Med*. 184, 465-472 (1996).
23. Sallusto, F. & Lanzavecchia, A. Dendritic cells use macropinocytosis and the mannose receptor to concentrate antigen in the MHC class II compartment. Downregulation by cytokines and bacte rial products. *J. Exp. Med*. 182, 389-400 (1995).
24. Jiang, W., et al. The receptor DEC-205 expressed by dendritic cells and thymic epithelial cells is involved in antigen processing. *Nature* 375, 151-155 (1995).
25. Kleijmeer, M.J., Oorschot, V.M.J. & Geuze, H.J. Human resident Langerhans cells display a lysosomal compartment enriched in MHC class II. *J. Invest. Dermatol*. 103, 516-523 (1994).
26. Kleijmeer, M.J., et al. MHC class II compartments and the kinetics of antigen presentation in activated mouse spleen dendritic cells. J. *Immunol*. 154, 5715-5724 (1995).
27. Nijman, H.W., et al. Antigen capture and MHC class II compartments of freshly isolated and cultured human blood dendritic cells. *J. Exp. Med*. 182, 163-174 (1995).
28. Steinman, R.M. The dendritic cell system and its role in immunogenicity. *Annu. Rev. Immunol*. 9, 271-296 (1991).
29. Caux C, Banchereau J. In vitro regulation of dendritic cell development and function. Blood Cell Biochemistry. Whetton T, Gordon J, editors. London: Plenum Press, 1996.
30.Williams, L.A., Egner, W.&Hart, D.N.J. Isolation and function of human dendritic cells. *Int. Rev. Cytol*. 153, 41-104 (1994).
29. Van Voorhis, W.C., et al. Relative efficacy of human monocytes and dendritic cells as accessory cells for T cell replication. *J. Exp. Med*. 158, 174-191 (1983).
30. Zvaifler, N.J., Steinman, R.M., Kaplan, G., Lau, L.L.&Rivelis, M. Identification of immunostimulatory dendritic cells in the synovial effusions of patients with rheumatoid arthritis. *J. Clin. Invest*. 76, 789-800 (1985).
31. Lang J, et al. Immunogenicity and safety of low-dose intradermal rabies vaccination given during an Expanded Programme on immunization session in Viet Nam: results of a comparative randomized trial.*Trans R Soc Trop Med Hyg* 93(2):208-13 (1999).
32. Tantawichien T, et al. Antibody response after a four site intradermal booster vaccination with cell-culutre rabies vaccine. Clin Inf Dis 28(5):1100-3 (1999).
33. Nagafuchi S, et al. Intradermal administration of viral vaccines. *Rev Med Virol* 8(2):97-111 (1998)
34. McClain, D.J.,et al. Immunologic Responses to Vaccinia Vaccines Administered by Different Parenteral Routes. *J Infect Dis* 175,756 –763 (1997).
34a. Tantawhichien T, et al. Antibody response after a four-site intradermal booster vaccination with cell cultures rabies vaccine. *Clin Inf Dis* 28:1100-3 (1999).
34b. Rahman F, et al. Cellular and humoral responses induced by intraderemal or intramuscular vaccination with the major hepatitis B surface antigen. *Hepatology* 31:512-7 (2000).
34c. Nirmal S, et al. Immune responses of infants to fractional doses of intradermally administered inactivated poliovirus vaccine. *Vaccine* 169:928-31 (1998).
35. Hsu, F.J., et al. Vaccination of patients with B-cell lymphoma using autologous antigen-pulsed dendritic cells. *Nat Med* 2, 52-58 (1996).
36. Nestle F, et al. Vaccination of melanoma patients with peptide- or tumor lysate-pulsed dendritic cells. *Nat Med* 4: 328-332 (1998).
37. Holtl, L, et al. CD83+ blood dendritic cells as a vaccine for immunotherapy of metastatic renal-cell cancer. *Lancet* 352: 1358 (1998).
38. Murphy, G.P., et al. Phase II Prostate Cancer Vaccine Trial: Report of a study involving 37 patients with disease recurrence following primary treatment. *Prostate* 39:54-59 (1999).
39. Austyn, J.M., Kupiec-Weglinski, J.W., Hankins, D.F. & Morris, P.J. Migration patterns of dendritic cells in the mouse. Homing to T cell-dependent areas of spleen, and binding within marginal zone. *J. Exp. Med*. 167, 646-651 (1988).
40. Fossum, S. Lymph-borne dendritic leucocytes do not recirculate, but enter the lymph node paracortex to become interdigitating cells. *Scand. J. Immunol*. 27, 97-105 (1989).

41. Kudo, S., Matsuno, K., Ezaki, T. & Ogawa, M. A novel migration pathway for rat dendritic cells from the blood: Hepatic sinusoids-lymph translocation. *J. Exp. Med*. 185, 777-84 (1997).
42. Inaba, K., et al. Identification of proliferating dendritic cell precursors in mouse blood. *J. Exp. Med*. 175, 1157-1167 (1992).
43. RidgeJ, Di Rosa P, Matziger P. A conditioned dendritic cell can be a temporal bridge between a CD4+ T helper and a T killer cell. *Nature* 393:474-8 (1998).
44. Barrat-Boyes, SM,Watkins SC, Finn, OJ. In vivo migration of dendritic cells differentiated in vitro: a chimpanzee model. *J Immunol*. 158:4543-7. (1997).
45. Bhardwaj, N., et al. Influenza virus-infected dendritic cells stimulate strong proliferative and cytolytic responses from human CD8+ T cells. *J. Clin. Invest*. 94, 797-807 (1994).
46. Bender, A., Bui, L.K., Feldman, M.A.V., Larsson, M. & Bhardwaj, N. Inactivated influenza virus, when presented on dendritic cells, elicits human CD8+ cytolytic T cell responses. *J. Exp. Med*. 182, 1663-1671 (1995).
47. Bhardwaj, et al. *Nature* 392 (6671):86-9 (1998).
48. Dhodapkar, MV, Steinman, RM, Sapp, M. Rapid generation of broad T-cell immunity in humans after a single injection of mature dendritic cells. *J Clin Invest*104:173-1880 (1999).
49. Moss B. Vaccinia virus: A tool for research and vaccine development. *Science* 252:1662-1667 (1991).
50. Haffar O., Garrigues J, Travis B, Moran P, Zarling J, Hu S-L. Human immunodeficiency virus-like, nonreplicating, *gag- env* particles assemble in a recombinant vaccinia virus expression system. *J Virol* ;64:2653-2659 (1990).
51.Excler J-L, Plotkin S. The prime-boost concept applied to HIV preventive vaccines. *AIDS* 11(suppl A):S127-137 (1997).
52. Redfield RR, Wright DC, James WD, Jones TS, Brown C, Burke DS. Disseminated vaccinia in a military recruit with human immunodeficiency virus (HIV) disease. *N Engl J Med* 316:673-676 (1987).
53. Baxby D, Paoletti E. Potential use of non-replicating vectors as recombinant vaccines. *Vaccine* 10:8-9 (1992).
54. Egan MA, Pavlat WA, Tartaglia J, et al. Induction of human immunodeficiency virus type 1 (HIV-1)-specific cytolytic T lymphocyte responses in seronegative adults by a nonreplicating, host-range-restricted canarypox vector (ALVAC) carrying the HIV-1MN env gene. *J Infect Dis* 171:1623-1627 (1995).
55. Fleury B, Janvier G, Pialoux G, et al. Memory cytotoxic T lymphocyte responses in human immunodeficiency virus type I (HIV-1)-negative volunteers immunized with a recombinant canarypox expressing gp160 of HIV-1 and boosted with a recombinant gp160. *J Infect Dis* 174:734-738 (1996).
56.Excler J-L, Plotkin S. The prime-boost concept applied to HIV preventive vaccines. *AIDS* 11(suppl A):S127-137 (1997).
57.Pialoux G, Excler J-L, Riviere L, et al. A prime-boost approach to HIV preventive vaccine using a recombinant canarypox virus expressing glycoprotein 160 (MN) followed by a recombinant glycoprotein 160 (MN/LAI). *AIDS Res Hum Retroviruses* 11:373-381 (1995).
58. Clements-Mann ML, et al. Immune responses to Human Immunodeiciency Virus (HIV) Type 1 induced by canarypox expressing HIV-1_{MN} gp120, HIV-1_{SF2} Recombinant gp120, or both vaccines in seronegative adults. *J Infect Dis* 177: 1230-46 (1998).
59. Belshe RB, et al. Induction of immune repsonses to HIV-1 by canarypox virus (ALVAC) HIV-1 and gp120SF-2 recombinant vaccines in uninfected volunteers. *AIDS* 12:2407-2415 (1998).
60. Salmon-Ceron D, et al. Safety and immunogenicity of a live recombinant canarypox virus expressing HIV type 1 gp120 MN tm/gag/protease LAI (ALVAC-HIV, vCP205) followed by a p24E-V3 MN synthetic peptide (CLTB-36) administered in healthy volunteers at low risk for HIV infection. *AIDS Res Hum Retroviruses* 15:633-45 (1999).

61. Evans TG, et al. A canarypox vaccine expressing multiple Human Immunodeficiency Virus type 1 genes given alone or with rgp120 elicits broad and durable CD8+ cytotoxic T lymphocyte responses in seronegative volunteers. *J Infect Dis* 180: 290-8 (1999).
62. Thurner B, et al. Vaccination with MAGE-3A1 peptide-pulsed mature, monocyte-derived dendritic cells expands specific cytotoxic T cells and induces regression of some metastases in advanced Stage IV melanoma. *J. Exp. Med*. 190:1669-78 (1999).
63. Dhodapkar MV and N. Bhardwaj. Active immunization of humans with dendritic cells. (manuscript submitted).
64. Cull G, et al. Generation of anti-idiotype immune responses following vaccination with idio-type protein pulsed dendritic cells in myeloma. *Brit J Haematology* 107:648-55 (1999).
65. Timmerman JM, et al. Linkage of foreign carrier protein or GM-CSF to a tumor antigen improves the efficacy of pulsed dendritic cell vaccination. *Blood* 90:514-8 (1997).
66. Engelmayer J, et al. Dendritic cells infected with canarypox induce anti-HIV CD8+ and CD4+ T cell responses from chronically infected individuals (manuscript submitted).
67. Ferrari G, et al. Clade B-based HIV-1 vaccines elicit cross-clade cytotoxic T lymphocyte reactivities in uninfected volunteers. *PNAS* 94:1396-401 (1997).
68. Keefer MC, et al. A Phase I trial of vaccinia-env/gag/pol (TCB-3B) given by alternative routes, boosted with rgp 120 MN. Int Conf AIDS 12:278 (abstract no. 496) 1998.

17. SIGNATURE PAGE

A Phase I study of Aventis Pasteur Live Recombinant ALVAC-HIV (vCP205, HIV-1 env/gag/pro) in Seronegative Adults Administered (1) Subcutaneously via *ex vivo* Transfected, Autologous Dendritic Cells (2) Intradermally, or (3) Intramuscularly.

I have read the foregoing protocol and agree to conduct the study as outlined. In addition, I agree to conduct the study in compliance with all applicable regulations and guidelines as stated in the protocol and other information supplied to me.

______________________________	________________________
**Principal Investigator's Signature**	**Date**

18. APPENDICES

18.1 Statement of Obligations of Sponsor/Monitor and Clinical Investigator

Sponsor/Monitor

The sponsor or his/her designated representative, the monitor, will:

1. Conduct a pre-investigation visit to:
    a. Establish the acceptability of the facility and record this in a written report (memorandum or form).

    b. Discuss the proposed clinical trial with the investigator, supply the case report form, the Investigator Brochure, and the draft protocol for review and approval.

    c. Discuss with the investigator FDA requirements with respect to informed consent, Institutional Review Board (IRB) approval of the trial, the protocol, including protocol amendments and informed consent changes.

2. Conduct periodic on-site visits to:
    a. Assure adherence to the protocol.

    b. Review case report forms and medical records for accuracy and completeness of information.

    c. Examine pharmacy records for documentation of: quantity and date of receipt of investigational vaccine, dispensation and accountability data for vaccine administration to each subject, loss of materials, contamination, etc, and unused supplies.

    d. Record, report (summarize) observations on the progress of the trial and continued acceptability of the facilities; prepare an on-site visit report.

    e. Review investigator files for required documents, i.e., protocols, protocol amendments, IRB approvals (protocols, amendments, informed consent, etc), IRB charter and membership, communications to and from the IRB and the monitor.

Clinical Investigator

1. Institutional Review Board (IRB)

    The investigator must assure the monitor that the IRB:

    a. Meets FDA regulations as defined in 21 CFR Part 56.

    b. Has authority delegated by the parent institution and found in IRB by-laws, operation guidelines or charter to approve, or disapprove, clinical trials and protocols including informed consent and other documents (protocol amendments, information to be supplied to subjects concerning informed consent, etc).

c. Complies with proper personnel makeup of IRB.

d. Convenes meetings using acceptable rules of order for making decisions, recording such decisions and implementing them.

e. Files contain (a) documentation of its decisions such as are found in IRB minutes and correspondence, (b) written guidelines or by-laws governing IRB functions, (c) protocols, (d) protocol information to be supplied to the subject, (f) correspondence between IRB and investigator (consent changes, protocol amendments, etc).

2. Informed Consent of Human Subjects.
The investigator must assure monitor that the informed consent for a subject:
a. Meets FDA regulations as defined in 21 CFR Part 50 Informed Consent.

b. Has been approved by the IRB, including, when required, information to be given to the subject regarding the trial he/she is enrolled in.
(1) Informed consent includes the basic elements and any additional elements necessary.
(2) The subject and a study site representative sign the form and the subject is given a copy.

3. Storage and Dispensing of Vaccine Supplies.
The investigator (or pharmacist) must assure (demonstrate to) the monitor that:
a. Adequate and accurate written records show receipt and disposition of all vaccine supplies, including dates, serial or lot number, quantities received, each quantity dispensed, administered or used with identification of each subject.

b. Purpose and reasons are given in written records for vaccine disposal, i.e., the amount contaminated, broken, or lost, etc, and quantity returned to the sponsor.

4. Case Report Forms.

The investigator must assure the monitor that:
a. Case report forms, when completed, accurately reflect the medical records on each subject or patient.

b. Case report forms and medical records will be accessible to the monitor or FDA inspectors' on-site visits.

5. Files and Records.

The investigator must assure the quality, integrity, and content of his/her files that will be inspected by the monitor and FDA inspectors. The files must contain, as a minimum:
a. Correspondence to and from IRB and the monitor.

b. Documents that include:
(1) IRB-approved protocols.
(2) IRB-approved protocol amendments.
(3) IRB-approved informed consent and information supplied to the subject or subject.
(4) IRB charter, membership, and their qualifications.

c. Documents and records must be retained for a minimum of 15 years.

b. Clinical supplies:
   (1) Record of receipt, date and quantity, batch or lot number.
   (2) Disposition dates and quantity administered to each subject.

6. Inventory records.

<u>Statement of Obligations and Responsibilities of the Medical monitor:</u>

The medical monitor is required to review all serious and unexpected adverse events (per ICH definitions) associated with the protocol and provide an unbiased written report of the event within 10 calendar days of the initial report. At a minimum, the medical monitor should comment on the outcomes of the adverse event (AE) and relationship of the AE to the test article. The medical monitor should also indicate whether he/she concurs with the details of the report provided by the study investigator.

18.2 TRIAL CONSENT FORM

(see attachment)

18.3 TEST OF UNDERSTANDING AND COMMITMENT

Test of Understanding and Commitment
RV 138

VCRC
Vaccine Clinical Research Center
1600 E. Gude Drive, Rockville. Tel: (301) 251-8351

Please circle true or false.

1	Participants in this vaccine study will definitely be protected against HIV	**T**	**F**
2	As part of this research study, participants in this vaccine study will get free HIV testing at the study site five times during the study.	**T**	**F**
3	Because the vaccines may turn some of the standard tests for HIV infection positive, some people may incorrectly think that the study participants are infected with HIV.	**T**	**F**
4	The vaccine tested in this study has already been used in over one thousand five hundred volunteers	**T**	**F**
5	A method used in this study requires exposing some of the volunteer's own blood cells to the vaccine and re-injecting them	**T**	**F**
6	Study participants may test positive for HIV antibodies and therefore may have problems getting life insurance, donating blood, traveling to a foreign country or joining the army	**T**	**F**
7	Some participants in this study will only get placebos	**T**	**F**
8	The main purpose of this study is to see if the method used to administer the vaccine is safe and stimulates the immune response	**T**	**F**
9	Participants in this vaccine study can get infected if they have unprotected sex or if they share needles	**T**	**F**
10	The placebos used in this vaccine study could cause side effects	**T**	**F**
11	Participants in this vaccine study could test HIV antibody positive, even though they are not infected with HIV	**T**	**F**
12	The commitment asked from participants in this vaccine study is for one year	**T**	**F**
13	The vaccine in this study uses a live canarypox virus containing HIV genes.	**T**	**F**
14	The vaccine in this study will give you HIV	**T**	**F**
15	Participants in this study vaccine can drop out if they choose to	**T**	**F**

Signature: ____ Date: _______________

One incorrect response is acceptable. The volunteer can get education about the misunderstanding and sign the Informed Consent form. If more than one response is incorrect, the participants will receive education and will take the TOU again at the same visit or at the next visit (for a maximum of 3 attempts to pass the test).

18.4 PREGNANCY REPORTING

For subjects who become pregnant after completing the third immunization: The subject may continue on the study with all scheduled visits, but no further immunizations will be given. For all subjects who become pregnant while on the trial (including those mentioned above), a Pregnancy Report CRF should be completed as soon as possible. The site should maintain contact with pregnant subjects to obtain pregnancy outcome information for the Pregnancy Follow-up CRF.

Specific information to be collected includes the following:

1. Pregnancy Report
   - Date of last menstrual period, date pregnancy confirmed, estimated date of confinement, history of children born with congenital abnormalities, and history of spontaneous abortions.

2. Pregnancy Follow-up

   - Date of delivery or termination, outcome of pregnancy (i.e., spontaneous abortion, therapeutic abortion, ectopic pregnancy, stillborn delivery, liveborn delivery) and the presence or absence of congenital abnormalities in the infant.

3. Abnormal Pregnancy Outcome-mother (To be completed in the event of a delivery of an infant with congenital abnormalities.)

   - Complications during pregnancy, labor and delivery; information regarding prenatal care, infections, illnesses, and medications taken during pregnancy; use of drugs, alcohol, or other conditions during the course of the pregnancy.

4. Abnormal Pregnancy Outcome-infant (To be completed in the event of a delivery of an infant with congenital abnormalities.)

   - The sex, weight, estimated gestational age, and description of abnormalities present in the infant.

18.5 MEDICAL MONITOR

COL. Nelson L. Michael, M. D.
Walter Reed Army Institute of Research
1600 E. Gude Drive
Rockville, MD 20850
W (301) 251-5051

18.6 Toxicity Grading Scale

TABLE FOR GRADING SEVERITY OF ADULT ADVERSE EXPERIENCES

PARAMETER	GRADE 1 MILD	GRADE 2 MODERATE	GRADE 3 SEVERE	GRADE 4 POTENTIALLY LIFE THREATENING
**HEMATOLOGY**				
Hemoglobin	9.5 g/dL - 10.5 g/dL	8.0 g/dL - 9.4 g/dL	7.9 g/dL - 6.5 g/dL	<6.5 g/dL
Absolute Neutrophil Count	1000 - 1500/mm3	750 - 999/mm3	500 - 749/mm3	<500/mm3
WBC	>13,000	>15,000	>20,000	>30,000 or <1,000
Percent Polys + Bands	>80%	90%	≥95%	_____
Platelets	100,000 - 120,000/mm3	75,000 - 99,999/mm3	50,000 - 74,999/mm3	20,000 - <50,000/mm3
CD4 Counts				
Uninfected	300 - 400/mm3<br><300 or <20%	300/mm3<br><200 or <18%	200/mm3<br><100 or 15%	<100/mm3<br><50 or <12%
Fibrinogen	100 - 200 mg/dL OR 400 - 600 mg/dL	<100 mg/dL OR >600 mg/dL	<50 mg/dL or associated with gross bleeding OR associated with disseminated coagulation	-----
Prothrombin Time (PT)	>1.0 - 1.24 x ULN	>1.25 - 1.49 x ULN	>1.5 - 3.0 x ULN	>3.0 x ULN
PTT	>1.0 - 1.66 x ULN	>1.66 - 2.33 x ULN	>2.33 - 3.0 x ULN	>3.0 x ULN
CHEMISTRIES				
CPK	≥4 ULN	≥6 ULN	≥10 ULN	≥20 ULN
Creatinine	>1.0 - 1.5 x ULN	>1.5 - 1.9 x ULN	>2.0 - 6.0 x ULN	>6.0 x ULN

SODIUM				
Hyponatremia	130 - 135 meq/L	123 - 129 meq/L	116 - 122 meq/L	<116 meq/L
Hypernatremia	146 - 150 meq/L	151 - 157 meq/L	158 - 165 meq/L	>165 meq/L
POTASSIUM				
Hyperkalemia	5.0 5.5 meq/L	5.6 - 6.0 meq/L	6.1 - 6.5 meq/L	>6.6 meq/L
Hypokalemia	3.2 - 3.4 meq/L	3.0 - 3.1 meq/L	2.5 - 2.9 meq/L	<2.5 meq/L
PHOSPHATE				
Hypophosphatemia	2.0 - 2.4 mg/dL	1.5 - 1.9 mg/dL	1.0 - 1.4 mg/dL	<1.0 mg/dL
CALCIUM (corrected for albumin)				
Hypocalcemia	7.8 - 8.4 mg/dL	7.0 - 7.7 mg/dL	6.1 - 6.9 mg/dL	<6.1 mg/dL
Hypercalcemia	10.6 - 11.5 mg/dL	11.6 - 12.5 mg/dL	12.6 - 13.5 mg/dL	>13.5 mg/dL
MAGNESIUM				
Hypomagnesemia	1.2 - 1.4 meq/L	0.9 - 1.1 meq/L	0.6 - 0.8 meq/L	<0.6 meq/L
BILIRUBIN				
Hyperbilirubinema	>1.0 - 1.5 x ULN	>1.5 - 2.5 x ULN	>2.5 - 5 x ULN	>5 x ULN
GLUCOSE				
Hypoglycemia	55 - 64 mg/dL	40 - 54 mg/dL	30 - 39 mg/dL	<30 mg/dL
Hyperglycemia (nonfasting and no prior diabetes)	116 - 160 mg/dL	161 - 250 mg/dL	251 - 500 mg/dL	>500 mg/dL
Triglycerides	-----	400 - 750 mg/dL	751 - 1200 mg/dL	>1200 mg/dL
URIC ACID				
Hyperuricemia	7.5 - 10.0 mg/dL	10.1 - 12.0 mg/dL	12.1 - 15.0 mg/dL	>15.0 mg/dL
LIVER TRANSAMINASE				
AST (SGOT)	1.51 - 2.5 x ULN	>2.5 - 5.0 x ULN	>5.0 - 10.0 x ULN	>10.0 x ULN
ALT (SGPT)	1.51 - 3.0 x ULN	>3.0 - 5.0 x ULN	>5.0 - 10.0 x ULN	>10.0 x ULN
GGT	1.51 - 2.5 x ULN	>2.5 - 5.0 x ULN	>5.0 - 10.0 x ULN	>10.0 x ULN
Alk Phos	1.51 - 2.5 x ULN	>2.5 - 5.0 x ULN	>5.0 - 10.0 x ULN	>10.0 x ULN

PANCREATIC ENZYMES				
Amylase	>1.0 - 1.5 x ULN	>1.5 - 2.0 x ULN	>2.0 - 5.0 x ULN	>5.0 x ULN
Pancreatic amylase	>1.0 - 1.5 x ULN	>1.5 - 2.0 x ULN	>2.0 - 5.0 x ULN	>5.0 x ULN
Lipase	>1.0 - 1.5 x ULN	>1.5 - 2.0 x ULN	>2.0 - 5.0 x ULN	>5.0 x ULN
CARDIO-VASCULAR				
Cardiac Arrhythmia	-----	Asymptomatic; transient dysrhythmia, no Rx req	Recurrent/persistent dysrhythmia; symptomatic Rx req	Unstable dysrhythmia, hospitalization and Rx req
Hypertension	Transient, increase >20 mm Hg diastolic BP; no Rx req	Recurrent; chronic increase >20 mm Hg diastolic BP; Rx req	Acute Rx req; outpatient OR hospitalization possible	Hospitalization req OR end organ damage
Hypotension	Transient orthostatic hypotension with heart rate increased by >20 beats/min OR decreased by <10 mm Hg systolic BP, no Rx req	Symptoms OR BP decreased by <20 mm Hg systolic, correctable with oral fluid Rx	IV fluid req OR hospitalization	Mean arterial pressure <60 mm HG OR end organ damage OR shock, vasopressor Rx req
Pericarditis	Minimal effusion	Mild/mod asymptomatic effusion, no Rx	Symptomatic effusion, pain, EKG changes	Tamponade OR pericardiocentesis OR surgery req
Hemorrhage, blood loss	-----	Mildly symptomatic, no Rx req	Gross blood loss OR 1-2 units transfused	Massive blood loss OR >2 unites transfused
Nausea	Mild OR transient; reasonable intake maintained	Mod discomfort OR intake decreased for <3 days	Severe discomfort OR minimal intake for ≥3 days	Hospitalization req

Vomiting	Mild OR transient; 2-3 episodes per day OR mild vomiting lasting <1 week	Mod OR persistent; 4-5 episodes per day; OR vomiting lasting ≥1 week	Severe vomiting of all food/fluids in 24 hrs OR orthostatic hypotension OR IV Rx req	Hypotensive shock OR hospitalization req for IV Rx req
Diarrhea	Mild OR transient, 3-4 loose stools per day OR mild diarrhea lasting <1 week	Mod OR persistent; 5-10 loose stools per day OR diarrhea lasting ≥ 1 week	>10 loose stools/day, bloody diarrhea; OR orthostatic hypotension OR electrolyte imbalance, >2 L IV fluid req	Hypotensive shock OR severe electrolyte imbalance
Oral Discomfort/Dysphagia	Mild discomfort, no difficulty swallowing	Difficulty swallowing but able to eat and drink	Unable to swallow solids	Unable to drink fluids; IV fluids req
Constipation		Moderate abdominal pain 78 hours with impaction require output prescription	Requiring disimpaction or hospital treatment	Distention with vomiting OR obstipation

RESPIRATORY

Cough (for aerosol studies)	Transient; no Rx	Treatment associated cough; inhaled bronchodilator	Uncontrolled cough; systemic Rx req	-----
Bronchospasm Acute	Transient; no Rx; FEV1 or peak flow reduced to 70% - 80%	Rx req; normalizes with bronchodilator; FEV1 or peak flow 50%-69%	No normalization with bronchodilator; FEV1 or peak flow 25%-49%, retractions	Cyanosis; FEV1 or peak flow <25% OR intubated
Dyspnea	Dyspnea on exertion	Dyspnea with normal activity	Dyspnea at rest	Dyspnea requiring O_2 therapy

NEUROLOGIC

Neuro-cerebellar	Slight incoordination OR dysdiadochokinesia	Intention tremor OR dysmetria OR slurred speech OR nystagmus	Ataxis requiring assistance to walk or arm incoordination interfering with ADLs	Unable to stand
Neuro-psych/mood	-----	-----	Severe mood changes requiring medical intervention; suicidal ideation	Acute psychosis req hospitalization; suicidal gesture/attempts
Paresthesia (burning, tingling, *etc*.)	Mild discomfort; no Rx req	Mod discomfort; non-narcotic analgesia req	Severe discomfort; OR narcotic analgesia req with symptomatic improvement	Incapacitating; OR not responsive to narcotic analgesia
Neuro-motor	Mild weakness in muscle of feet but able to walk and/or mild increase or decrease in reflexes	Mod weakness in feet (unable to walk on heels and/or toes), mild weakness in hands, still able to do most hand tasks and/or loss of previously present reflex or development of hyperreflexia and/or unable to do deep knee bends due to weakness	Marked distal weakness (unable to dorsiflex toes or foot drop, and mod proximal weakness *e.g.*, in hands interfering with ADLs and/or req assistance to walk and/or unable to rise from chair unassisted	Confined to bed or wheel chair because of muscle weakness

Neuro-sensory	Mild impairment (decreased sensation, *e.g.*, vibratory, pinprick, hot/cold in great toes) in focal area or symmetrical distribution	Mod impairment (mod decreased sensation, *e.g.*, vibratory, pinprick, hot/cold to ankles) and/or joint position or mild impairment that is not symmetrical	Severe impairment (decreased or loss of sensation to knees or wrists) or loss of sensation of at least mod degree in multiple different body areas (*i.e.*, upper and lower extremities)	Sensory loss involves limbs and trunk
**MUSCULO-SKELETAL**				
Arthralgia/Arthritis	Arthralgia	Arthralgia with joint effusion or moderate impairment of activity	Frank arthritis with or without effusion OR resulting in severe impairment of activity	-----
Myalgia	Myalgia without limitation of activity	Muscle tenderness at other than injection site or with moderate impairment of activity	Frank myonecrosis OR with severe impairment of activity	-----
**CUTANEOUS**				
Rash/Dermatitis	Erythema, pruritus	Diffuse maculopapular rash OR dry desquamation	Vesiculation OR moist desquamation OR ulceration	ANY ONE: mucous membrane involvement, suspected Stevens-Johnson (TEN), erythema multiforme, necrosis req surgery, exfoliative dermatitis

Local Reaction	Erythema OR induration <15 x 15 cm (225 cm^2)	Erythema, induration, or edema >15 x 15 cm (225 cm^2)	Ulceration OR super infection OR phlebitis	Necrosis of the skin
**URINALYSIS**				
Proteinuria<br>Random urine	1+	2-3+	4+	Nephrotic syndrome
24 Hour Urine	200 mg - 1 g loss/day OR <0.3% OR <3 g/L	1-2 g loss/day OR 0.3 - 1.0% OR 3-10 g/L	2-3.5 g loss/day OR >1.0% OR >10 g/L	Nephrotic syndrome OR >3.5 g loss/day
Hematuria	Microscopic only £10 rbc/hpf	>10 rbc/hpf	Gross, with or without clots OR RBC casts	Obstructive OR transfusion req
**MISCELLANEOUS**				
Fever<br>oral >12 hours	37.7 - 38.9C (100.0 - 101.5F)	39.0 - 39.5C (101.6 - 102.9F) OR max temp of 103F	39.6 - 40.5C (103 - 105F) OR max temp of 103.5F	>40.5C (105F) OR max temp >105F
Headache	Mild, no Rx req, OR non-narcotic analgesia Rx	Mod; OR responds to initial narcotic Rx	Severe; intractable; OR req repeated narcotic Rx	Req hospitalization, associated with neurologic, respiratory or cardiovascular abnormalities
Allergic Reaction	Pruritus without rash at injection site	Localized urticaria at injection site	Generalized urticaria angioedema	Anaphylaxis
ADL	Normal activity reduced <48 hours	Normal activity reduced 25%-50% >48 hours	Normal activity reduced >50%; cannot work >48 hours	Unable to care for self

EYE	Mild pain, visual changes, conjunctival erythema, abnormal slit lamp	Loss of vision, clinically diagnosed uveitis, moderate to severe pain, glaucoma	-----

ADL = Activities of Daily Living
